# Real‐World Weight Loss Is Associated With a Reduction in Cancer Risk

**DOI:** 10.1002/oby.70163

**Published:** 2026-03-10

**Authors:** Kenda Alkwatli, Huijun Xiao, Arshiya Mariam‐Smith, Nerea Lopetegui, Marcio L. Griebeler, Bartolome Burguera, Kevin M. Pantalone, Daniel M. Rotroff

**Affiliations:** ^1^ Endocrinology Starling Physicians Wethersfield Connecticut USA; ^2^ Center for Quantitative Metabolic Research Cleveland Clinic Cleveland Ohio USA; ^3^ Department of Endocrinology and Metabolism Medical Specialty Institute, Cleveland Clinic Cleveland Ohio USA; ^4^ Department of Quantitative Health Sciences Cleveland Clinic Research, Cleveland Clinic Cleveland Ohio USA; ^5^ Comprehensive Cancer Center The Ohio State University Columbus Ohio USA

**Keywords:** obesity/complications, real‐world data, risk reduction behavior

## Abstract

**Objective:**

Obesity is a major risk factor for multiple cancers, yet the impact of nonsurgical weight loss on cancer risk remains uncertain. Our objective was to evaluate whether real‐world nonsurgical weight loss is associated with a reduced risk of developing cancer.

**Methods:**

We conducted a retrospective observational study of 143,630 adults (BMI ≥ 30) from an integrated US health system's electronic health records (2000–2022). Cases with incident cancer were compared to matched controls. Using generalized linear models with a logit link, we assessed whether BMI change over 3‐, 5‐, and 10‐year intervals is associated with the risk of diagnosis for obesity‐related and other cancers.

**Results:**

Among 143,630 patients, 7703 cases and 135,927 controls were identified. Each 1% BMI reduction was linked to lower obesity‐related cancer risk at 3 years (OR, 0.990; 95% CI, 0.984–0.996; *p* < 0.001), 5 years (OR, 0.989; 95% CI, 0.984–0.995; *p* < 0.001), and 10 years (OR, 0.992; 95% CI, 0.984–1.000; *p* = 0.057). Similar associations were observed for other cancer types across all intervals (OR < 1; *p* < 0.001).

**Conclusions:**

Real‐world weight loss was associated with a decreased risk of obesity‐related and other cancers.

## Study Importance

- What is already known?
  ○ Obesity is a well‐established risk factor for multiple types of cancer, but the impact of nonsurgical real‐world weight loss on cancer risk remains unclear.
- What does this study add?
  ○ This study shows that even modest real‐world weight loss is associated with significantly reduced odds of both obesity‐related and other cancers over 3‐, 5‐, and 10‐year intervals.
- How might these results change the direction of research or the focus of clinical practice?
  ○ These findings emphasize the importance of implementing effective weight loss interventions as part of cancer prevention strategies in routine clinical care.

## 1 Introduction

Obesity is a chronic disease associated with many comorbidities, including cancer [1]. Thirteen cancers have been strongly linked with obesity, including esophageal adenocarcinoma, gastric cardia, colon and rectum, liver, gallbladder, pancreas, postmenopausal breast, endometrium, and ovarian cancer, renal cell carcinoma, meningioma, thyroid cancer, and multiple myeloma [2]. Obesity is thought to increase the risk of cancer through multiple interrelated mechanisms, including hormonal dysregulation, chronic inflammation, and adipokine imbalance [3, 4, 5, 6, 7, 8, 9, 10, 11, 12, 13, 14, 15, 16, 17].

Moreover, excess body weight has been proposed to be the second greatest preventable risk factor of cancer after smoking in both males and females [18]. Now, with the development of highly effective antiobesity medications (AOMs), new avenues for medical management of obesity are emerging.

Weight loss following bariatric surgery has been associated with a decreased risk of cancer [19]. The intensive clinical monitoring involved in presurgical programs and postsurgical follow‐up visits provides rich data to evaluate the impact of bariatric surgery on cancer risk. In contrast, observational studies examining the association between cancer and nonsurgical weight loss have been small and report conflicting results, possibly due to insufficient power or a short time frame for follow‐up [20, 21, 22]. Prospective studies tracking nonsurgical weight loss through conventional methods, such as lifestyle modification, over long periods are challenging because they require extended follow‐up, substantial resources, and high patient adherence. Electronic health records (EHRs) offer an alternative, providing a large real‐world database suitable for case–control studies investigating the associations between weight loss and cancer. Here, we aim to investigate the extent to which obesity‐related cancer risk may be reduced by real‐world weight loss in a patient population captured from a large integrated health system.

## 2 Methods

### 2.1 Patient Cohort

Following approval from the Institutional Review Board, patients seen at Cleveland Clinic between January 1, 2000, and December 31, 2022, were included in the study. Patients who were at least 20 years old with a body mass index (BMI) > 30 kg/m^2^ and at least seven visits over 3 years were included. The age criterion was set at ≥ 20 years, as height typically stabilizes by this age, allowing for more accurate calculation of percent weight change. Patients with a history of alcohol or substance abuse and dependence, amputations, HIV, organ transplant, and thyroid problems at baseline were excluded from the cohort based on International Classification of Diseases, Ninth Revision (ICD‐9) and International Classification of Diseases, Tenth Revision (ICD‐10) codes (Table S1). Individuals with bariatric surgery were also excluded based on ICD (ICD‐10: Z98.84 and ICD‐9: V45.86) and Current Procedural Terminology (CPT) codes (43644, 43645, 43659, 43770–43775, 43845–43848, 43886‐43888, 43842, 43843).

Individuals with no documented history of cancer were eligible as controls, and those with a documented cancer diagnosis were assessed for inclusion as cases based on the criteria described here.

### 2.2 Cancer Diagnosis

Patient data across 25 distinct cancer endpoints, including two composite endpoints—obesity‐related cancers and all cancers—across multiple follow‐up intervals, were included for analysis. Only new cancer diagnoses were included, and individuals with a prior cancer diagnosis or fewer than two distinct contact encounters for a given diagnosis were excluded.

Each endpoint was evaluated in three separate cohorts based on weight change over time intervals of 3, 5, or 10 years preceding the cancer diagnosis. The primary and secondary endpoints are described here.

#### 2.2.1 Obesity‐Related Cancers (Primary Endpoints)

Obesity‐related cancer endpoints included primary cancers of the esophagus, liver, gallbladder, pancreas, colon, rectum, kidney (i.e., renal cell carcinoma), and endometrium, multiple myeloma, and postmenopausal breast cancer based on ICD‐9 and ICD‐10 diagnosis codes listed in Table S2a. Additionally, a composite of these cancers was also considered a primary endpoint.

Cancers of the thyroid and gastric cardia had insufficient statistical power, defined as power ≤ 0.7, or had zero cases at specific time intervals and were subsequently excluded from the analysis.

#### 2.2.2 All Cancers (Secondary Endpoints)

All malignant neoplasm diagnosis codes (including primary endpoints) categorized according to the ICD‐10 hierarchy were examined, including (i) lip, oral cavity, and pharynx, (ii) digestive organs, (iii) respiratory and intrathoracic organs, (iv) melanoma and other malignant neoplasms of skin, (v) mesothelial and soft tissue, (vi) breast, (vii) female genital organs, (viii) male genital organs, (ix) urinary tract, (x) eye, brain, and other parts of the central nervous system, (xi) malignant neoplasms of ill‐defined, secondary, and unspecified sites, (xii) lymphoid, hematopoietic, and related tissue, (xiii) neuroendocrine tumors. A composite endpoint of all listed malignancies was also assessed.

A list of ICD‐9 and ICD‐10 codes of cancers included in secondary endpoints is provided in Table S2b.

### 2.3 Data Processing

#### 2.3.1 BMI Change (Percentage)

**FIGURE 1:**
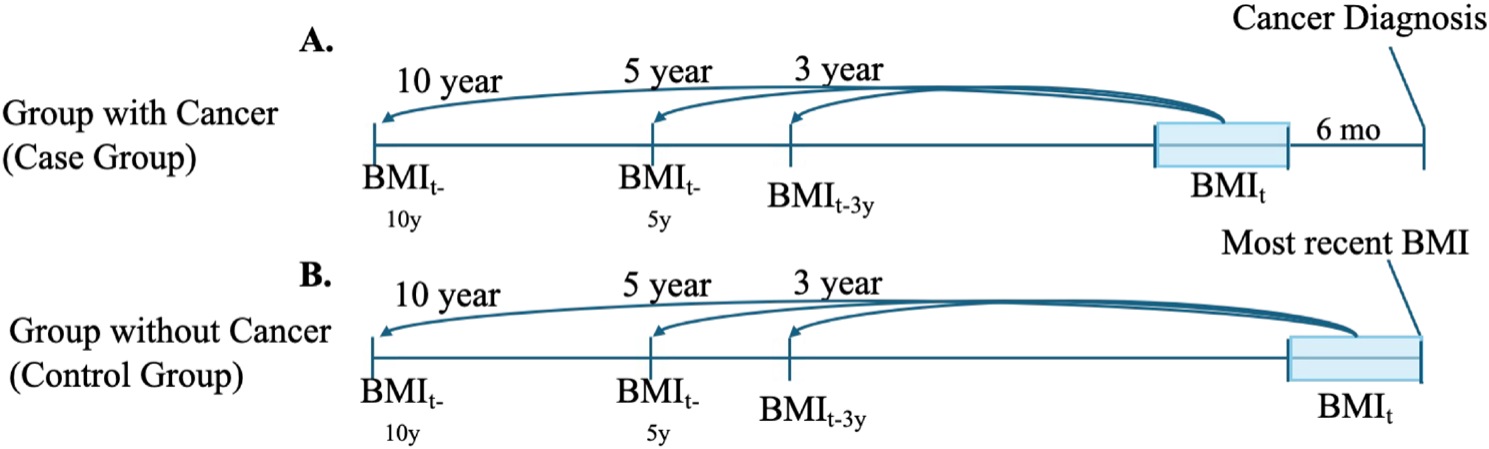
Calculation of BMI change in those with and without cancer diagnosis. (A) Group with cancer (case group) and (B) group without cancer (control group). [Color figure can be viewed at wileyonlinelibrary.com]

We investigated percentage changes in BMI over 3, 5, and 10 years. For patients with a cancer diagnosis (cases), BMI values measured 6–12 months before cancer diagnosis were compared with BMI values from 3, 5, and 10 years prior (Figure 1). BMI measurements obtained more than 6 months and up to 12 months before diagnosis were used to reduce potential bias from cancer‐related weight fluctuations. If multiple BMI measurements were available within the 6– to 12‐month window, the value closest to 6 months before diagnosis was used. For patients with no cancer diagnosis (controls), BMI values were compared between the last visit and values from 3, 5, and 10 years prior. If a BMI measurement was missing at a specified time point (3, 5, or 10 years prior), the closest available measurement within a ±6‐month range of that time point was used. Thus, index dates for cases were defined as 3, 5, and 10 years before cancer diagnosis and for controls as 3, 5, and 10 years before their last BMI measurement.

#### 2.3.2 Other Variables

Type 2 diabetes (T2D) was determined using a modified version of the eMERGE algorithm described in Kho et al. [23, 24]. This algorithm utilizes ICD‐9, ICD‐10, clinical labs, and medications to identify patients with T2D and exclude patients with type 1 diabetes.

Age, gender, race, state of residence, smoking status, and medication use (including diabetes medications, antihypertensive medications, lipid‐lowering medications, medications for depression, and hormonal therapy for menopause) were analyzed at the start of the follow‐up period. More specific information on these variables is included in Table S3a–c.

### 2.4 Statistical Analysis

#### 2.4.1 Balancing Cases and Controls Using Inverse Probability Weighting (IPW)

To ensure comparability between case and control groups within each cohort, IPW was employed using *WeightIt* [25]. Patients in the control group were younger than the case group (9 years difference in median age). IPW weights were used in subsequent regression analyses to address age‐related weight change between cases and controls. This method allowed for the balancing of the cohorts by assigning weights to each participant based on their probability of assignment to either the case or the control group, enhancing the validity of the comparative analyses. Trimming of the weighted data was performed after IPW to exclude outliers and reduce the influence of extreme weights, thus refining the cohorts further. Only the trimmed, weighted cohorts with clipped weights were utilized in the final analyses. This represented a robust approach by allowing the same control group to be used for comparisons with multiple endpoints, and this IPW approach has been previously used for nested case–control studies [26].

#### 2.4.2 Demographics and Baseline Differences

Differences in patient characteristics, such as demographics, comorbidities, and medication classes, between cases and controls were described as follows. Continuous variables were tested for normality using the Shapiro–Wilk tests, histogram plots, and QQ plots, as appropriate. Continuous variables with normal distribution were tested using two‐sample *t*‐tests to assess the difference between cases and controls. Continuous variables that showed signs of nonnormality based on distribution plots were tested using the Wilcoxon rank sum test. Pearson's *χ* ^2^ test or Fisher's exact test was used for testing categorical variables. Univariable analysis was conducted using logistic regression models to assess the association between patient characteristics and cancer endpoints. Variables that showed significance in the univariable analysis were considered for inclusion as covariates in subsequent association testing.

#### 2.4.3 Association Testing

Separate generalized linear models with a logit link function, incorporated with IPW, were used to assess the association between BMI changes at 3, 5, and 10 years before diagnosis and each cancer endpoint. Key baseline covariates were included in the models: BMI, age, gender, race, smoking history, Elixhauser comorbidity index, Charlson comorbidity index, and state of residence.

Assessments for multicollinearity among potential covariates were conducted using the variance inflation factor (VIF). Only covariates with VIF value < 3 were included in the final models. Pairwise Pearson correlations among continuous covariates were low to moderate, with the highest correlation observed between Elixhauser and Charlson indexes (*r* = 0.70), consistent with their overlapping constructs (Table S4). Together with VIF value < 3, these findings indicate no evidence of problematic multicollinearity. Variables were excluded from the analyses if they had high missingness, group imbalance, or high correlation with other variables. In cases where the cohort was imbalanced, the Firth adjustment was applied. Endpoints with insufficient power (i.e., power smaller than the threshold of 0.7) in all three time points analyses or endpoints with no cases were dropped. All models were diagnosed and checked using appropriate residual plots. Model fit was assessed using the Hosmer‐Lemeshow test, and all models demonstrated adequate fit (all *p* > 0.05). Residual and influence diagnostics (standardized residuals, leverage, and Cook's distance) were further examined, and no observations were identified as exerting undue influence. Data were managed and analyzed using R software (version 4.4.1, Vienna, Austria). Final models for all cancer endpoints and follow‐up periods were then adjusted for multiple comparisons using the Benjamini–Hochberg method to control the false discovery rate (FDR). For purpose of transparency, uncorrected *p* values were also reported; however, conclusions regarding statistical significance should be obtained from the FDR *p* values. The overall significance threshold was a two‐sided α of less than 0.05.

#### 2.4.4 Sensitivity Analyses

The two composite endpoints were used to perform sensitivity analyses. Odds ratios (OR) before and after IPW adjustment were compared to evaluate the impact of weighting. Given that individuals with a Charlson comorbidity index (CCI) are more likely to experience unintentional weight loss [27], subgroup analyses by CCI were conducted to assess whether effect sizes were consistent across comorbidity levels. Additional subset analyses were performed by gender (female vs. male). Further stratification of individual cancer types was not pursued given insufficient case numbers and limited power, making the composite endpoints the most reliable and representative for subgroup evaluation.

## 3 Results

Out of 525,873 adults seen at Cleveland Clinic during the study period, 143,630 met the inclusion criteria (Figure S1). Baseline demographics and patient characteristics over 3‐, 5‐, and 10‐year cohorts are summarized in Table 1A, 1B, 1C. The sample size was smaller for longer follow‐up periods (3 years: 115,942, 5 years: 105,472, 10 years: 59,112). Approximately 3.6% to 6.0% of these cohorts were cases. During all three time intervals, the majority of the patients were White (78%), with mean ages ranging from 50 to 55 years old (Table 1A, 1B, 1C). Compared to controls, the cases were older and had higher comorbidity burden (*p* < 0.05). Compared to cases, a greater percentage of controls were taking GLP‐1RA and SGLT‐2i medications during all follow‐up periods (*p* < 0.001). Baseline differences in BMI between the cases and controls were observed for the 3‐ and 5‐year follow‐ups, highlighting the importance of including baseline BMI in the analysis. The median weight change for the complete cohort was close to no weight change (0%) (Figure S2). The weight change distribution was shifted lower for the cases compared to the controls (Figure S3).

**TABLE 1A:** Patient characteristics in 3‐year cohort.

Characteristic	Overall *N* = 115,942^a^	Controls *N* = 108,969^a^	Cases *N* = 6973^a^	*p* ^b^
Age	55.00 (43.00, 66.00)	55.00 (42.00, 66.00)	64.00 (55.00, 72.00)	**< 2.2e−16**
Gender				**< 2.2e−16**
Female	60,342 (52%)	57,385 (53%)	2957 (42%)	
Male	55,600 (48%)	51,584 (47%)	4016 (58%)	
Race				**< 2.2e−16**
Black	22,604 (19%)	21,570 (20%)	1034 (15%)	
Others	2802 (2.4%)	2690 (2.5%)	112 (1.6%)	
White	90,536 (78%)	84,709 (78%)	5827 (84%)	
State of residence				**0.008**
Florida	8359 (7.2%)	7908 (7.3%)	451 (6.5%)	
Ohio	99622 (86%)	93,621 (86%)	6001 (86%)	
Other US state	7961 (6.9%)	7440 (6.8%)	521 (7.5%)	
BMI at event	34.37 (31.63, 38.77)	34.39 (31.63, 38.85)	34.19 (31.75, 37.66)	**1.57e−04**
BMI at 3 years prior	34.50 (31.91, 38.77)	34.50 (31.89, 38.84)	34.38 (31.99, 37.90)	**0.005**
Smoking history				**< 2.2e−16**
Current	19,401 (17%)	19,399 (18%)	2 (< 0.1%)	
Former	27,750 (24%)	24,462 (22%)	3288 (47%)	
Never	67,575 (58%)	63,954 (59%)	3621 (52%)	
Passive	1216 (1.0%)	1154 (1.1%)	62 (0.9%)	
Type 2 diabetes	24,383 (21%)	22,349 (21%)	2034 (29%)	**< 2.2e−16**
Charlson comorbidity index	0.00 (0.00, 1.00)	0.00 (0.00, 1.00)	1.00 (0.00, 2.00)	**< 2.2e−16**
Elixhauser comorbidity Index	1.00 (1.00, 2.00)	1.00 (1.00, 2.00)	2.00 (1.00, 2.00)	**< 2.2e−16**
GLP‐1RA	7555 (6.5%)	7306 (6.7%)	249 (3.6%)	**< 2.2e−16**
Biguanides	31,656 (27%)	29,516 (27%)	2140 (31%)	**5.86e−11**
Loop diuretics	27,260 (24%)	25,015 (23%)	2245 (32%)	**< 2.2e−16**
Phentermine	45 (< 0.1%)	45 (< 0.1%)	0 (0%)	0.11
SGLT‐2i	6028 (5.2%)	5859 (5.4%)	169 (2.4%)	**< 2.2e−16**
Thiazide diuretics	47,856 (41%)	44,349 (41%)	3507 (50%)	**< 2.2e−16**

*Note*: The bold values indicate the categories.

^a^ Median (IQR); *n* (%).

^b^ Wilcoxon rank sum test; Pearson's *χ* ^2^ test; Fisher's exact test.

**TABLE 1B:** Patient characteristics in 5‐year cohort.

Characteristic	Overall *N* = 105,472^a^	Controls *N* = 100,143^a^	Cases *N* = 5,329^a^	*p* ^b^
Age	54.00 (41.00, 65.00)	53.00 (41.00, 64.00)	62.00 (53.00, 70.00)	**< 2.2e−16**
Gender				**< 2.2e−16**
Female	54,810 (52%)	52,541 (52%)	2269 (43%)	
Male	50,662 (48%)	47,602 (48%)	3060 (57%)	
Race				**< 2.2e−16**
Black	20,826 (20%)	20,044 (20%)	782 (15%)	
Others	2531 (2.4%)	2438 (2.4%)	93 (1.7%)	
White	82,115 (78%)	77,661 (78%)	4454 (84%)	
State of residence				**0.003**
Florida	7398 (7.0%)	7075 (7.1%)	323 (6.1%)	
Ohio	91,286 (87%)	86,662 (87%)	4624 (87%)	
Other US state	6788 (6.4%)	6406 (6.4%)	382 (7.2%)	
BMI at event	34.48 (31.70, 38.92)	34.50 (31.70, 38.97)	34.25 (31.79, 37.82)	**5.90e−04**
BMI at 5 years prior	34.57 (31.94, 38.79)	34.57 (31.94, 38.83)	34.51 (32.10, 38.24)	0.29
Smoking history				**< 2.2e−16**
Current	17,881 (17%)	17,881 (18%)	0 (0%)	
Former	24,955 (24%)	22,494 (22%)	2461 (46%)	
Never	61,532 (58%)	58,712 (59%)	2820 (53%)	
Passive	1104 (1.0%)	1056 (1.1%)	48 (0.9%)	
Type 2 diabetes	22,702 (22%)	21,121 (21%)	1,581 (30%)	**< 2.2e‐16**
Charlson comorbidity index	0.00 (0.00, 1.00)	0.00 (0.00, 1.00)	1.00 (0.00, 2.00)	**< 2.2e‐16**
Elixhauser comorbidity index	1.00 (1.00, 2.00)	1.00 (1.00, 2.00)	2.00 (1.00, 2.00)	**< 2.2e‐16**
GLP‐1RA	7254 (6.9%)	7024 (7.0%)	230 (4.3%)	**3.37e‐14**
Biguanides	29,613 (28%)	27,924 (28%)	1689 (32%)	**1.62e‐09**
Loop diuretics	24,684 (23%)	22,928 (23%)	1756 (33%)	**< 2.2e‐16**
Phentermine	41 (< 0.1%)	41 (< 0.1%)	0 (0%)	0.27
SGLT‐2i	5837 (5.5%)	5688 (5.7%)	149 (2.8%)	**< 2.2e‐16**
Thiazide diuretics	44,583 (42%)	41,788 (42%)	2,795 (52%)	**< 2.2e‐16**

*Note*: The bold values indicate the categories.

^a^ Median (IQR); *n* (%).

^b^ Wilcoxon rank sum test; Pearson's *χ* ^2^ test; Fisher's exact test.

**TABLE 1C:** Patient characteristics in 10‐year cohort.

Characteristic	Overall *N* = 59,112^a^	Controls *N* = 56,975^a^	Cases *N* = 2,137^a^	*p* ^b^
Age	50.00 (39.00, 60.00)	50.00 (39.00, 60.00)	58.00 (50.00, 66.00)	**< 2.2e−16**
Gender				**7.07e−10**
Female	30,753 (52%)	29,781 (52%)	972 (45%)	
Male	28,359 (48%)	27,194 (48%)	1165 (55%)	
Race				**5.55e−12**
Black	11,763 (20%)	11,461 (20%)	302 (14%)	
Others	1369 (2.3%)	1332 (2.3%)	37 (1.7%)	
White	45,980 (78%)	44,182 (78%)	1798 (84%)	
State of residence				**0.008**
Florida	4040 (6.8%)	3929 (6.9%)	111 (5.2%)	
Ohio	51,791 (88%)	49,890 (88%)	1901 (89%)	
Other US state	3281 (5.6%)	3156 (5.5%)	125 (5.8%)	
BMI at event	34.87 (31.93, 39.27)	34.88 (31.93, 39.33)	34.41 (31.93, 37.85)	**3.16e−04**
BMI at 10 years prior	34.61 (31.98, 38.73)	34.61 (31.98, 38.73)	34.57 (32.03, 38.15)	0.33
Smoking history				**< 2.2e−16**
Current	9806 (17%)	9806 (17%)	0 (0%)	
Former	13,610 (23%)	12,665 (22%)	945 (44%)	
Never	35,077 (59%)	33,905 (60%)	1172 (55%)	
Passive	619 (1.0%)	599 (1.1%)	20 (0.9%)	
Type 2 diabetes	13,756 (23%)	13,076 (23%)	680 (32%)	**< 2.2e−16**
Charlson comorbidity index	0.00 (0.00, 1.00)	0.00 (0.00, 1.00)	1.00 (0.00, 2.00)	**< 2.2e−16**
Elixhauser comorbidity index	1.00 (1.00, 2.00)	1.00 (1.00, 2.00)	2.00 (1.00, 2.00)	**< 2.2e−16**
GLP‐1RA	4990 (8.4%)	4882 (8.6%)	108 (5.1%)	**9.59e−09**
Biguanides	18,230 (31%)	17,468 (31%)	762 (36%)	**9.02e−07**
Loop diuretics	14,001 (24%)	13,271 (23%)	730 (34%)	**< 2.2e−16**
Phentermine	35 (< 0.1%)	35 (< 0.1%)	0 (0%)	0.64
SGLT‐2i	3981 (6.7%)	3896 (6.8%)	85 (4.0%)	**2.22e−07**
Thiazide diuretics	27,226 (46%)	26,036 (46%)	1190 (56%)	**< 2.2e−16**

*Note*: The bold values indicate the categories.

^a^ Median (IQR); *n* (%).

^b^ Wilcoxon rank sum test; Pearson's *χ* ^2^ test; Fisher's exact test.

### 3.1 Obesity‐Related Cancers (Primary Endpoints)

**FIGURE 2:**
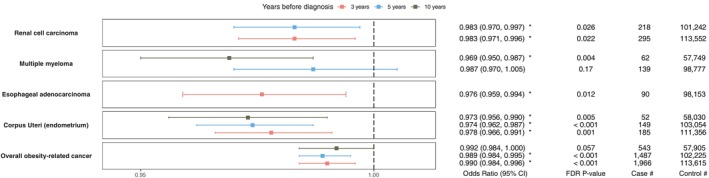
Forest plot of BMI decrease OR with 95% CI, FDR‐adjusted *p* value, and number of cases and controls in adjusted multivariable analysis for each primary cancer endpoint (*obesity‐related cancers*). BMI change with a significant *p* value in final models was marked with *. Renal cell carcinoma at 10 years, multiple myeloma at 3 years, and esophageal cancer at 5 and 10 years were excluded from analysis due to insufficient statistical power. [Color figure can be viewed at wileyonlinelibrary.com]

After FDR adjustment, 5 of 11 primary endpoints were associated with weight change (Figure 2 and Table S5). The risk of developing overall obesity‐related cancers was lower in those with weight loss at 3 years (OR = 0.990; 95% CI, 0.984–0.996) and 5 years (OR = 0.989; 95% CI, 0.984–0.995). This translated to 4.9% and 5.4% reduction in odds for 5% weight loss at 3 and 5 years, respectively. Consistent with the complete cohorts, the association between weight change and the overall obesity cancer endpoint remained significant for the 3‐ and 5‐year time intervals in the CCI < 3 subsets (FDR *p* < 0.05) (Table S7). However, no significant associations were observed for the CCI > 3 subset. Weight loss was associated with the overall obesity cancer endpoint for the 3‐year follow‐up period in both males and females (OR < 0.991, FDR *p* < 0.05) (Table S8).

Different patterns emerged in the associations between specific cancer types and weight change. Weight loss was associated with lower odds of endometrial cancer at 3‐, 5‐, and 10‐year follow‐ups (OR < 0.978, FDR *p* < 0.05) (Figure 2). Similarly, it was linked to reduced odds of renal cell carcinoma at 3 and 5 years (OR = 0.983, FDR *p* < 0.05). In contrast, only weight loss over the 10‐year interval was associated with lower odds of developing multiple myeloma (OR = 0.969, 95% CI, 0.950–0.987). Results for all obesity‐related cancers are provided in Table S4.

### 3.2 All Cancers (Secondary Endpoints)

**FIGURE 3:**
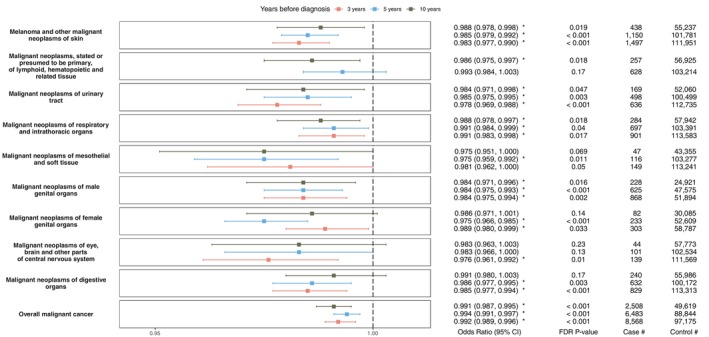
Forest plot of BMI decrease OR with 95% CI, FDR‐adjusted *p* value, and number of cases and controls in adjusted multivariable analysis for each secondary cancer endpoint (*all cancers*). BMI changes with significant *p* values in final models were marked with *. Malignant neoplasms, stated or presumed to be primary, of lymphoid, hematopoietic, and related tissue at 3 years were excluded from analysis due to insufficient statistical power. [Color figure can be viewed at wileyonlinelibrary.com]

**FIGURE 4:**
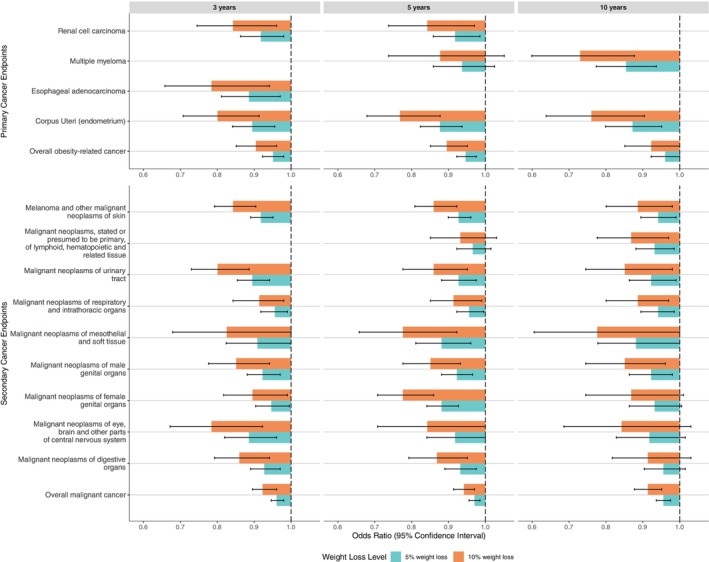
OR of 5% and 10% weight loss at the 3‐, 5‐, 10‐year time intervals for primary and secondary cancer endpoints with statistical significance. [Color figure can be viewed at wileyonlinelibrary.com]

Additionally, 10 of 12 secondary endpoints were associated with weight change (Figure 3, Table S6). Our results demonstrated that the odds of developing any malignancy were lower in those who lost weight over 3 years (OR = 0.992; 95% CI, 0.989–0.996), 5 years (OR = 0.994; 95% CI, 0.991–0.997), and 10 years (OR = 0.991; 95% CI, 0.987–0.995) (Figure 3). Individuals with 5% weight loss had 3.9%, 3.0%, and 4.4% lower odds of developing any malignancy at 3, 5, and 10 years, respectively (Figure 4). Weight loss over a time interval as short as 3 years was associated with lower odds of developing seven secondary endpoints, including malignant neoplasms of the digestive system (OR < 0.989, FDR *p* < 0.05).

Weight loss was significantly associated with lower odds of developing any malignant cancer in the CCI < 3 subgroup across all time intervals (Table S7). Similar associations were observed in the CCI ≥ 3 subgroup at the 3‐ and 5‐year intervals. In both males and females, weight loss was associated with lower odds of developing any malignancy during all three follow‐up periods (OR < 0.993, FDR *p* < 0.05) (Table S8).

The results from the sensitivity analysis demonstrated that the unweighted results were consistent with the weighted findings, demonstrating the same directionality and similar magnitudes. These results support that the conclusions are robust and not driven by the weighting approach (Table S9).

## 4 Discussion

Obesity rates have tripled over the past two decades [28, 29, 30, 31], and sufficient evidence demonstrates that excess body weight significantly increases the risk of developing multiple cancers [2, 28, 29, 30]. In this study of 143,630 individuals, real‐world weight loss among individuals with obesity was inversely associated with the risk of obesity‐related cancers and all malignancies over 3, 5, and 10 years of follow‐up (Figures 1, 2, 3).

Statistical significance was observed at different time points for renal cell carcinoma, multiple myeloma, esophageal cancer, and endometrial cancer, as well as in other cancers, including melanoma and other malignant neoplasms of the skin, malignant neoplasms of lymphoid, hematopoietic and related tissues, malignant neoplasms of the urinary tract, respiratory and intrathoracic organs, mesothelial and soft tissue, male genital organs, female genital organs, eye, brain, and other parts of the central nervous system, and digestive organs (FDR *p* < 0.05).

Several mechanisms may underlie the link between obesity and cancer development, and these mechanisms may differ across cancer types and include impacts on insulin sensitivity and the immune microenvironment [32, 33]. Increased adipose tissue mass disrupts the production of adiponectin, resistin, leptin, and free fatty acids [3, 4, 34], contributing to hyperinsulinemia and insulin resistance, both of which are implicated in cancer pathogenesis [5].

Increased binding of insulin‐to‐insulin receptors has been shown to directly promote tumor growth of gastric cancer cells and has implications for clinical management of patients with obesity, diabetes, and cancer [6, 7, 8]. Leptin also stimulates proliferation of the colon epithelium and inhibits apoptosis [14]. Elevated serum levels associated with obesity have also been linked to gastric cancer progression [15]. Although our study was not powered to specifically evaluate gastric cancer, we observed lower odds of developing malignancies of the digestive organs for individuals with weight loss (3‐year OR = 0.985, 5‐year OR = 0.986, FDR *p* < 0.05).

Specifically, reduced risk of endometrial cancer and renal cell carcinoma with weight loss was observed for three and two follow‐up intervals, respectively. Obesity disrupts sex hormones through increased insulin resistance. Previous in vitro and in vivo studies show that these hormone disruptions are associated with modified risk of breast cancer [26, 27, 28], endometrial cancer [13], and renal cell carcinoma [23]. Restoration of immune surveillance and T cell function and normalization of adipokines after weight loss are particularly relevant for hormonally driven cancers (e.g., breast, endometrial) [35, 36] and those with strong immune involvement (e.g., colorectal) [37].

Increased risk of multiple myeloma with weight gain was also observed. This is consistent with previous reports of adipocyte accumulation in bone marrow leading to microenvironment changes promoting multiple myeloma progression [16, 17]. While preclinical studies have reported increased risk of breast cancer due to the disruption of sex hormones, breast cancer risk was not associated with weight change in our study. However, this lack of association is consistent with the results of the SPLENDID trial of bariatric surgery [38]. A more complex interplay between obesity and life stage may account for this as early‐life obesity and postmenopausal weight changes have been associated with altered risk of breast cancer [28, 38]. Future mechanistic studies integrating longitudinal data and multiomics are warranted to clarify the mechanisms attenuating the risk and identify potential drug targets [39, 40].

Emerging evidence suggests that weight loss following bariatric surgery is associated with a reduced risk of cancer [41, 42, 43, 44]. For example, Aminian et al. analyzed EHR data from 30,318 patients with a median follow‐up of 6.1 years and reported a 2% absolute risk difference of obesity‐related cancer cumulative incidence (95% CI, 1.2%–2.7%) between patients who had bariatric surgery and controls, with similar results for both Roux‐en‐Y and gastric sleeve techniques [19]. However, evidence for nonsurgical weight loss is sparse. Moore et al. pooled data from 12 prospective cohorts examining 26 cancers and found that high levels of leisure‐time physical activity were associated with lower risks of developing 13 types of cancers [45]. Our study leveraged a cohort of 143,630 individuals and suggests that real‐world nonsurgical weight loss can modify cancer risk. Although intentional weight loss is not directly captured in the EHR, the associations remained significant in analyses adjusted for comorbidities and sensitivity analyses by CCI subgroups indicate that each 1% change in BMI is associated with significantly reduced odds of developing cancer (Figures 1, 2, 3).

We now have the emergence of effective AOMs that can cause meaningful weight loss, some similar to bariatric surgery results [46, 47, 48]. A retrospective observational study by Wang et al. suggested a decreased risk of obesity‐related cancers (except thyroid and postmenopausal breast cancer) with GLP‐1RA compared with insulins in patients with T2D [38]. Together with our findings on real‐world weight loss among individuals with obesity, these results highlight the importance of investigating the mediating role of GLP‐1RA induced weight loss in cancer risk among individuals with obesity. There is a need for future prospective studies to assess whether AOMs could modify the risk of cancer in patients with obesity and whether it is through weight loss or independent mechanisms.

5

Our study has several limitations that must be considered when interpreting the findings. Despite the relatively long period of follow‐up and efforts for comprehensively assessing baseline characteristics, as well as the use of a casual interference approach to normalize cases and controls, as with any study, there remains the possibility of potential confounding factors that could have influenced the results. As the inclusion in the study required sufficient longitudinal EHR data, individuals with higher health care utilization may have been more likely to be included. This could introduce selection bias, as the study population may not be fully representative of the general population. Since the last BMI measurement in controls did not precede a specific documented disease diagnosis, it is unlikely to have been influenced by disease‐related changes in weight.

Here, we studied real‐world weight loss, and due to limitations in the EHR, we are unable to differentiate between intentional and nonintentional weight loss. However, our previous study by Mariam et al. demonstrated that adjusting for prior comorbidities helped adjust for perceived issues with unintentional weight loss [24]. Additionally, weight change was measured as a change in BMI, which has its limitations, as it is unable to distinguish between lean and fat mass. Our study also did not address whether weight loss was maintained over the entire follow‐up period or what impact weight variability has on cancer risk. The modeling approach used assumes a linear relationship between weight change and risk. Future work should investigate the existence of nonlinear relationships between weight change and risk of cancer.

Furthermore, due to the study's observational nature, we could not assess whether weight loss preceding cancer diagnosis was related to delay in diagnosis. However, we expect this potential limitation was mitigated by excluding BMI measurements reported within 6 months preceding cancer diagnosis.

Despite these limitations, our study has multiple strengths as it offers one of the largest cohorts in a real‐world integrated health system, in addition to comprehensive, detailed longitudinal clinical data with a follow‐up period up to 10 years.

## 6 Conclusion

Real‐world weight loss was associated with a decreased risk of developing obesity‐related cancers and all other cancers. Our study serves as a call for action and a strong public health message to health care stakeholders to intensify efforts and resources to treat obesity as a chronic disease to help reduce the risk of developing cancer.

We now have powerful and effective medications and other tools for weight loss that can help treat obesity as a chronic disease and maintain weight loss. These advancements provide new hope for patients suffering from obesity; however, further research is essential to understand their long‐term effects on cancer risk and the mechanisms behind their success.

## Author Contributions

All authors contributed to the conceptualization of the study. H.X., A.M.‐S., and D.M.R. were responsible for data curation and analysis. K.A., H.X., A.M.‐S., and D.M.R. drafted the manuscript. K.M.P., N.L., M.L.G., and B.B. provided supervision, reviewed the manuscript, and offered revisions and suggestions. All authors read and approved the final manuscript.

## Funding

The study was funded in part by the Cleveland Clinic Center for Quantitative Metabolic Research.

## Conflicts of Interest

D.M.R. reports research funding from Bayer Pharmaceuticals and Novo Nordisk; a license agreement with OpenDNA for diabetes stratification; consulting fees from Genovation Health LLC and Clarified Precision Medicine; speaker honoraria from the International Society for Anesthetic Pharmacology; and being an inventor on patents related to precision treatment recommendations and GLP‐1RA responsiveness (International Patent Application No. PCT/US2022/28611 and US Provisional Patent Application No. 63/737,152). He holds leadership roles as Chief Technology Officer at Clarified Precision Medicine and Chief Medical Information Officer at Genovation Health and owns equity in both companies. K.M.P. reports receiving institutional consulting and research support from Twin Health (paid to Cleveland Clinic) and personal consulting fees from Bayer, Boehringer Ingelheim, Corcept Therapeutics, Diasome, Eli Lilly, Merck, Novo Nordisk, and Sanofi. K.M.P. has received payment or honoraria for lectures, presentations, speakers bureaus, manuscript writing, or educational events from AstraZeneca, Corcept Therapeutics, and Novo Nordisk. In addition, K.M.P. has a pending patent application titled Identifying Patients for Intensive Hyperglycemia Management (US Provisional Patent Application No. 62/982,195). A.M. has patent applications related to intensive management of diabetes (US Provisional Patent Application No. 62/982,195) and response to GLP‐1RA treatments (International Patent Application No. PCT/US2022/28611 and US Provisional Patent Application No. 63/737,152). The other authors declare no conflicts of interest.

## Supporting information


**Data S1:** oby70163‐sup‐0001‐Supinfo.docx.

## Data Availability

The data that support the findings of this study are available from the corresponding author upon reasonable request.
