# Supplementary material for: Real‐World Weight Loss Is Associated With a Reduction in Cancer Risk
Source: Obesity (Silver Spring). 2026 Mar 10;34(4):928–38. doi: 10.1002/oby.70163 (PMC13032047; doi:10.1002/oby.70163)
Supplement: Supplementary file 1 — Data S1: oby70163‐sup‐0001‐Supinfo.docx. [file OBY-34-928-s001.docx]

**Supplementary Material**

**Real-World Weight Loss is Associated with a Reduction in Cancer Risk**

Kenda Alkwatli^1,2,3^, Huijun Xiao^2,4^, Arshiya Mariam-Smith^2,4^, Nerea Lopetegui^2,5^, Marcio L. Griebeler^2,3^, Bartolome Burguera^2,3^, Kevin M. Pantalone^2,3,4^, Daniel M. Rotroff ^2,3,4,#^

1. Endocrinology Starling Physicians Endocrinology, Wethersfield, CT, USA

2. Center for Quantitative Metabolic Research, Cleveland Clinic, Cleveland, OH, USA

3. Department of Endocrinology and Metabolism, Medical Specialty Institute, Cleveland Clinic, Cleveland, OH, USA

4. Department of Quantitative Health Sciences, Cleveland Clinic Research, Cleveland Clinic, Cleveland, OH, USA

5. Comprehensive Cancer Center, The Ohio State University, Columbus, OH, USA

#**Corresponding Author Contact Information:**

Daniel M. Rotroff, PhD, MSPH,

Department of Quantitative Health Sciences, Cleveland Clinic Research,

Cleveland Clinic,

9500 Euclid Avenue, JJN3-01,

Cleveland, OH 44195, United States

[rotrofd@ccf.org](mailto:rotrofd@ccf.org)

**Figures**

Figure S1. Flowchart


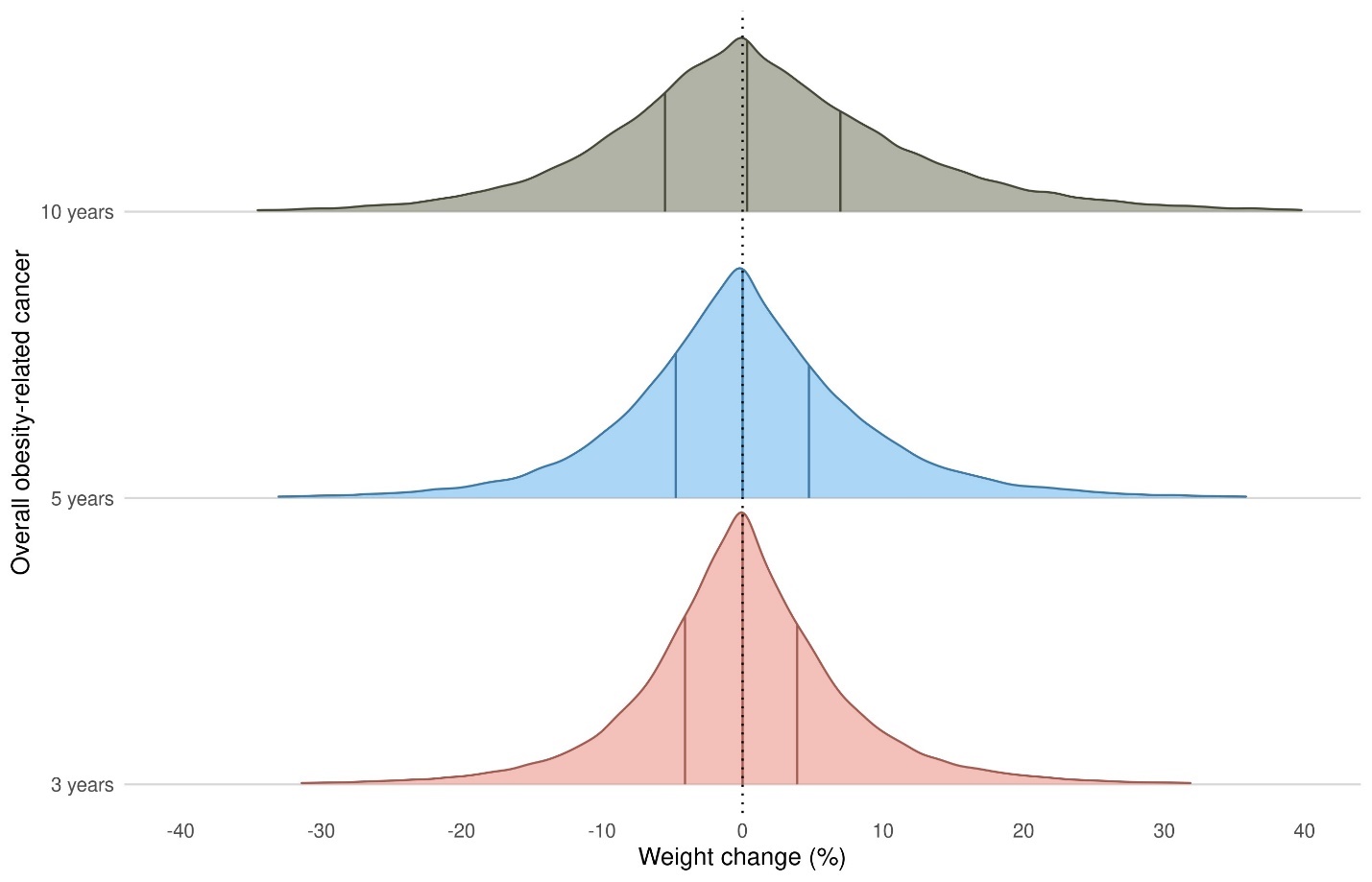


Figure S2: Distribution of Overall Percentage Weight Change for Obesity-Related Cancers


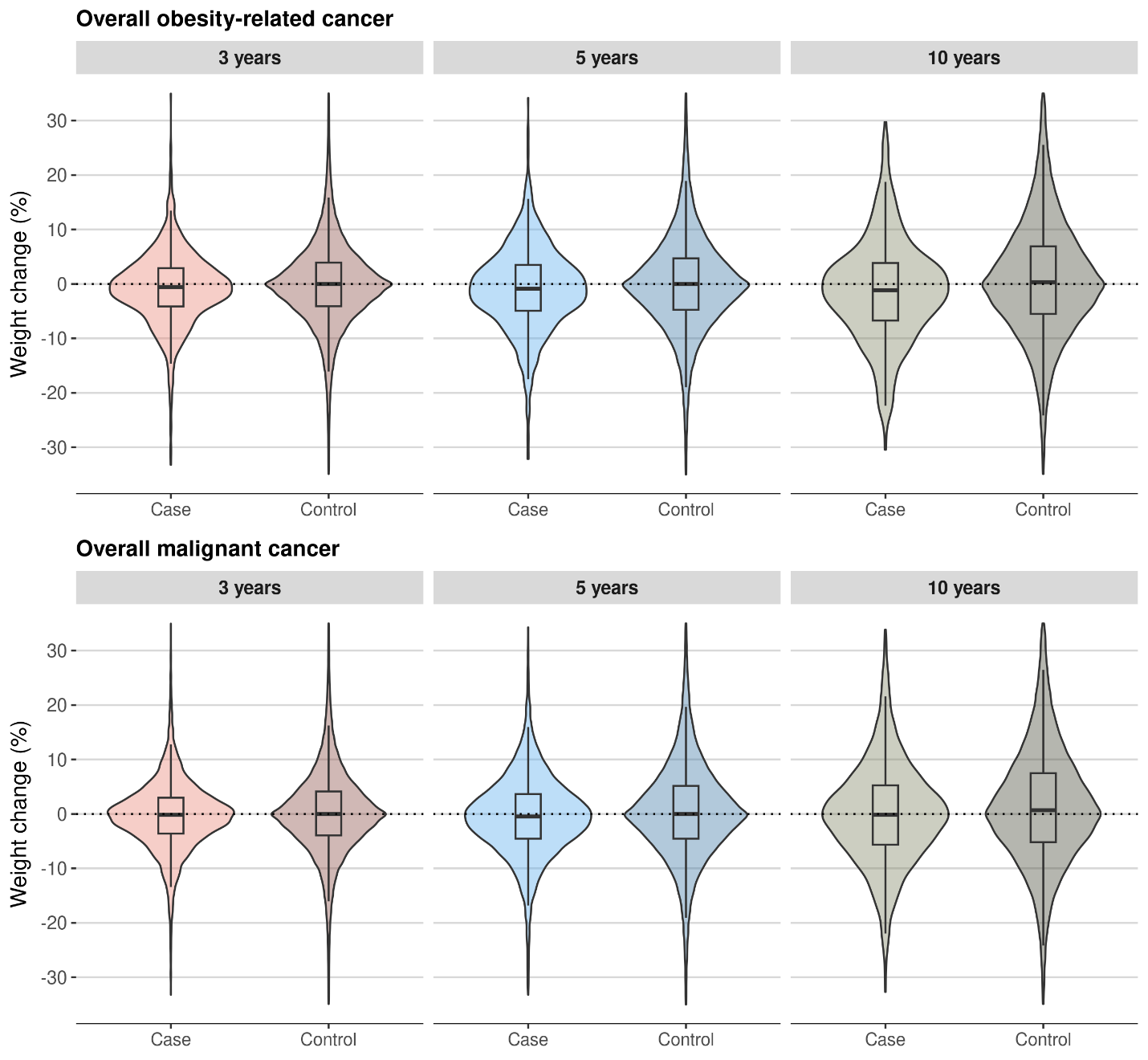


Figure S3: Distribution of Weight Change (%) in Case and Control Groups for

**Tables**

| **Table S1. List of ICD codes used in the exclusion criteria** | |
| --- | --- |
| **Comorbidities** | **Codes** |
| HIV | **ICD9:** 042  **ICD10:** B20 |
| Thyroid Problems | **ICD9:** 240, 241, 242, 243, 245, 246  **ICD10:** E00, E01, E02, E03, E04, E05, E06, E07 |
| Amputations | **ICD9:** NA  **ICD10:** Z89 |
| Organ Transplant | **ICD9:** V420, V421, V423, V424, V426, V427, V428  **ICD10:** Z940, Z941, Z942, Z943, Z944, Z945, Z946, Z948 |
| Alcohol Abuse and Dependence | **ICD9:** 303  **ICD10:** NA |
| Substance Abuse | **ICD9:** 292  **ICD10:** F11, F12, F13, F14, F15, F16, F18, F19 |

| **Table S2a. Diagnosis Codes Used to Assist in Identifying Malignant Obesity-Related Cancers (Primary Endpoints)** | |
| --- | --- |
| **Diagnosis** | **Codes** |
| Postmenopausal | **ICD9:** 174.0, 174.1, 174.2, 174.3, 174.4, 174.5, 174.6, 174.7, 174.8, 174.9  **ICD10:** C50.01, C50.011, C50.012, C50.019, C50.11, C50.111, C50.112, C50.119, C50.21, C50.211, C50.212, C50.219, C50.31, C50.311, C50.312, C50.319, C50.41, C50.411, C50.412, C50.419, C50.51, C50.511, C50.512, C50.519, C50.61, C50.611, C50.612, C50.619, C50.81, C50.811, C50.812, C50.819, C50.91, C50.911, C50.912, C50.919 |
| Esophageal adenocarcinoma | **ICD9:** 150.0, 150.1, 150.2, 150.3, 150.4, 150.5, 150.8, 150.9  **ICD10:** C15.3, C15.4, C15.5, C15.8, C15.9 |
| Gastric cardia | **ICD9:** 151.0  **ICD10:** C16.0 |
| Liver | **ICD9:** 155.0, 155.1, 155.2  **ICD10:** C22.0, C22.1, C22.2, C22.3, C22.4, C22.7, C22.8, C22.9 |
| Gallbladder | **ICD9:** 156.0, 156.8  **ICD10:** C23 |
| Pancreas | **ICD9:** 157.0, 157.1, 157.2, 157.3, 157.4, 157.8, 157.9  **ICD10:** C25.0, C25.1, C25.2, C25.3, C25.4, C25.7, C25.8, C25.9 |
| Colon | **ICD9:** 153.0, 153.1, 153.2, 153.3, 153.4, 153.5, 153.6, 153.7, 153.8, 153.9  **ICD10:** C18.0, C18.1, C18.2, C18.3, C18.4, C18.5, C18.6, C18.7, C18.8, C18.9 |
| Rectum | **ICD9:** 154.0, 154.1, 154.8  **ICD10:** C19, C20 |
| Renal Cell carcinoma | **ICD9:** 189.0  **ICD10:** C64.1, C64.2, C64.9 |
| Ovary | **ICD9:** 183.0, 183.8, 183.9  **ICD10:** C56.1, C56.2, C56.9 |
| Corpus Uteri (endometrium) | **ICD9:** 182.0, 182.1, 182.8  **ICD10:** C54.0, C54.1, C54.2, C54.3, C54.8, C54.9 |
| Thyroid | **ICD9:** 193  **ICD10:** C73 |
| Multiple myeloma | **ICD9:** 203.00, 203.01, 203.02  **ICD10:** C90.00, C90.01, C90.02 |

| **Table S2b. Diagnosis Codes Used to Assist in Identifying Any Type of Malignant Cancer (Secondary Endpoints)** | |
| --- | --- |
| **Diagnosis** | **Codes** |
| Malignant neoplasm of breast | **ICD9:** 174, 175  **ICD10:** C50 |
| Malignant neoplasms of bone and articular cartilage | **ICD9:** 170  **ICD10:** C40,C41 |
| Malignant neoplasms of digestive organs | **ICD9:** 150, 151, 152, 153, 154, 155, 156, 157, 159  **ICD10:** C15, C16, C17, C18, C19, C20, C21, C22, C23, C24, C25, C26 |
| Malignant neoplasms of eye, brain and other parts of central nervous system | **ICD9:** 190, 191, 192  **ICD10:** C69, C70, C71, C72 |
| Malignant neoplasms of female genital organs | **ICD9:** 180, 181, 182, 183, 184  **ICD10:** C51, C52, C53, C54, C55, C56, C57, C58 |
| Malignant neoplasms of ill-defined, secondary and unspecified sites | **ICD9:** 195, 196, 197, 198, 199  **ICD10:** C76, C77, C78, C79, C80 |
| Malignant neoplasms of lip, oral cavity and pharynx | **ICD9:** 140, 141, 142, 143, 144, 145, 146, 147, 148, 149  **ICD10:** C00, C01, C02, C03, C04, C05, C06, C07, C08, C09, C10, C11, C12, C13, C14 |
| Malignant neoplasms of male genital organs | **ICD9:** 185, 186, 187  **ICD10:** C60, C61, C62, C63 |
| Malignant neoplasms of mesothelial and soft tissue | **ICD9:** 158, 176, 171  **ICD10:** C45, C46, C47, C48, C49 |
| Malignant neoplasms of respiratory and intrathoracic organs | **ICD9:** 160, 161, 162, 163, 164, 165  **ICD10:** C30, C31, C32, C33, C34, C35, C36, C37, C38, C39 |
| Malignant neoplasms of thyroid and other endocrine glands | **ICD9:** 193, 194  **ICD10:** C73, C74, C75 |
| Malignant neoplasms of urinary tract | **ICD9:** 188, 189  **ICD10:** C64, C65, C66 ,C67, C68 |
| Malignant neoplasms, stated or presumed to be primary, of lymphoid, hematopoietic and related tissue | **ICD9:** 200, 201, 202, 203, 204, 205, 206, 207, 208, 23879  **ICD10:** C81, C82, C83, C84, C85, C86, C87, C88, C89, C90, C91, C92, C93, C94, C95, C96 |
| Melanoma and other malignant neoplasms of skin | **ICD9:** 172,173  **ICD10:** C43,C44 |
| Neuroendocrine tumors | **ICD9:** 209  **ICD10:** C7A,C7B |
| Malignant neoplasms of independent (primary) multiple sites | **ICD9:** NA  **ICD10:** C97 |

| **Table S3a. Concept IDs for Medication Classes under Medications for Depression** | |
| --- | --- |
| **Class** | **Concept ID** |
| Adrenergic alpha-Antagonists | C0595230, C0595231, C4019582, C0979913, C0979917, C0991767, C1276909, C1699036, C1699037, C1949280, C1949283, C1949285, C2702061, C2731595, C2731596 |
| Adrenergic alpha1-Antagonists | C0703031, C0703032, C0703033, C0978918, C1168677, C4704350, C4704355, C0713361, C0713362, C1509556, C0939127, C0939128, C0874811, C0690610, C0589987, C1306044, C2740386, C2740390, C2740393, C2740395 |
| Adrenergic alpha2-Antagonists | C4704350, C4704355, C0713361, C0713362, C1509556, C0939127, C0939128 |
| Cholinergic Muscarinic Antagonists | C4704350, C4704355, C0713361, C0713362, C1509556, C0939127, C0939128 |
| Cytochrome P450 2D6 Inhibitors | C3249485, C3249491, C3857250, C3857220, C1711760, C1812859, C2316499, C2683197, C2316500, C2609731, C2316502, C2609733, C0874618 |
| Dopamine Agonists | C3162605, C3162607, C3162611, C3162613, C4033645, C4033056, C4033644, C4033055, C4033643, C4033054, C4033642, C4033053, C4033641, C4033052, C4033640, C4033051, C1965986, C1966465, C1269858, C0715287, C1269859, C0715288, C0979718, C0716263, C0981691, C0715291, C0715290, C2741606, C2741609, C2741613, C2741615, C2741618, C2741619, C2741622, C2741623, C2741627, C2741629 |
| Dopamine Antagonists | C0595230, C0595231, C4019582, C0687887, C0687888, C0687889, C0687890, C0979913, C0979917, C0991767, C1276909, C1699036, C1699037, C1949280, C1949283, C1949285, C2702061, C2731595, C2731596 |
| Dopamine Uptake Inhibitors | C1509298, C1585896, C1652555, C0978582, C1509299, C1169579, C1169800, C1169840, C0939654, C1169842, C0939108, C1509993, C1509569, C1586272, C0361632, C1623488, C0876573, C0689571, C0708399, C3249485, C3249491, C3499670, C3499676, C3857250, C3857220, C4018714, C4018717, C4018719, C4018723, C4018726, C4018729, C4018732, C4018735, C4059353, C4059172, C4080549, C4080551, C4080560, C4080562, C4489866, C4489873, C4489875, C4489877, C4489879, C4489881, C4541939, C4719436, C5140558, C5139977, C5140559, C5139978, C5140560, C5139979, C5140561, C5139980, C5140562, C5139981, C5140563, C5139982, C5136870, C5135773, C5136869, C5135772, C5136868, C5135771, C5136867, C4745580, C5136866, C5135770, C5679093, C5679094, C5679136, C5679137, C1695074, C1711742, C1695954, C1711744, C1711746, C1711748, C1711760, C1812859, C2316499, C2683197, C2316500, C2609731, C2316502, C2609733, C0874618 |
| GABA A Modulators | C0355015, C0355054, C0707632, C0542887, C0591006, C3833271, C3856255, C0689399, C1169995, C0689400, C0690437, C0690438, C0690439, C0717695, C0692687, C0693721, C0708005, C0708012, C0710044, C0710045, C0710046, C4765159, C4765164, C0717163, C5553559, C5553567, C5553569, C5553571, C5553573, C5553576, C5576961, C5576964, C0974161, C0978249, C1576507, C1584697, C1585611, C1585612, C1329641, C1329640, C1329639, C1329638 |
| Glycine Agonists | C3833271, C3856255, C1585611, C1585612 |
| Hormone Receptor Agonists | C2980897, C2980999, C3265506, C3265561, C3281374, C3281376, C3464113, C3464932, C3464939, C3484870, C3528622, C3642688, C3644521, C3666794, C3668737, C3882758, C4019787, C4058261, C4238183, C4238187, C4306112, C4523012, C4523051, C0692069, C0692493, C0692505, C0692506, C0692915, C2106240, C0692916, C0693220, C0717519, C0693232, C0693233, C0693785, C0693956, C0694082, C5245337, C5416893, C5416894, C0780277, C5440230, C0794896, C0795005, C0795007, C0795395, C5441842, C0873597, C5578618, C5578767, C5578768, C0874140, C0937448, C2683351, C2722832, C2725104 |
| Monoamine Oxidase Inhibitors | C0355297, C0716260, C0355299, C0355300, C0698295, C0979431, C0980782, C0692794, C0713593, C1724003, C1724006, C0980121, C1698388, C1696454, C1700019, C1697519, C1698387, C1696453, C0733060, C0355760, C2724939, C1962764, C1962766, C1962763, C2732172, C1962765 |
| Noncompetitive NMDA Receptor Antagonists | C4762644, C4762650, C4762651, C4762652, C4762653, C4762654 |
| Norepinephrine Transporter Interactions | C0717518, C1655464, C1169992, C0689387, C2073821, C0692744, C0693401, C0707965, C0792947, C0792948, C0978225, C1966345, C1967316, C0689381, C2683249 |
| Norepinephrine Uptake Inhibitors | C0688670, C0976542, C0976541, C0874810, C0710930, C1509298, C1585896, C1652555, C0978582, C1509299, C1169579, C1169800, C1169840, C0939654, C1169842, C0939108, C1509993, C1509569, C1586272, C0361632, C1623488, C0876573, C0689571, C0708399, C0703031, C0703032, C0703033, C0978918, C1168677, C0688473, C0706344, C0688474, C0706345, C0688475, C0706348, C0688476, C0706350, C0706351, C0688478, C0706353, C3249485, C3249491, C0355266, C0689429, C0689430, C0689431, C3499670, C3499676, C3663526, C3663532, C3663534, C3663536, C3663538, C3663540, C3663543, C3663545, C3663548, C3665175, C3665177, C3857250, C3857220, C4018221, C4018714, C4018717, C4018719, C4018723, C4018726, C4018729, C4018732, C4018735, C4026301, C4059353, C4059172, C4080549, C4080551, C4080560, C4080562, C4317352, C4317350, C4489866, C4489873, C4489875, C4489877, C4489879, C4489881, C0687887, C0687888, C0687889, C0687890, C0689818, C0689819, C0692697, C0692887, C0692963, C0692964, C4541939, C2146612, C0693480, C4719436, C0710471, C0711085, C0711098, C0711111, C0711156, C5140558, C5139977, C5140559, C5139978, C5140560, C5139979, C5140561, C5139980, C5140562, C5139981, C5140563, C5139982, C5136870, C5135773, C5136869, C5135772, C5136868, C5135771, C5136867, C4745580, C5136866, C5135770, C5207667, C5207500, C5207668, C5207501, C5207669, C5207502, C5207670, C5207503, C5384142, C5384143, C5384144, C5384145, C0791893, C5679093, C5679094, C5679136, C5679137, C5679138, C0979061, C0980855, C0980857, C0980858, C0980962, C0980963, C0980964, C0980967, C0981790, C0991723, C1351193, C1509241, C1656295, C1532660, C0716075, C1695074, C1711742, C1695954, C1711744, C1711746, C1711748, C0361135, C2341613, C2341630, C2317356, C2316847, C2608681, C2608684, C2608686, C2608688, C2608689, C2608691, C2608692, C2608694, C2608695, C0981532, C0689184, C0689188, C0689187, C0355262, C0689186, C0689185, C0689181, C0874811, C0690610, C0589987, C1306044, C0981208, C0974280, C0687872, C0974281, C0715047, C0981269, C0698280, C0687874, C0687875, C1997479, C0786990, C0542923, C0688370, C0698288, C0688371, C0698289, C0688372, C0707125, C0787445, C0793359, C2730277, C2730281, C2731196, C2731200, C2731202, C2731204, C2731206, C0690210, C0690211, C2747641, C2747645, C2747647, C2747649, C1711760, C1812859, C2316499, C2683197, C2316500, C2609731, C2316502, C2609733, C0874618 |
| Serotonin 5HT-3 Antagonists | C3667587, C3667588, C3667589, C3667611, C4238276, C4238280, C4238282, C4704350, C4704355, C0713361, C0713362, C1509556, C0939127, C0939128, C0874811, C0690610, C0589987, C1306044, C2740386, C2740390, C2740393, C2740395 |
| Serotonin Agonists | C0692873, C0688071, C0542894, C1169245, C0688072, C0355063, C0937519 |
| Serotonin Antagonists | C0595230, C0595231, C4019582, C0979913, C0979917, C0991767, C1276909, C1699036, C1699037, C1949280, C1949283, C1949285, C2702061, C2731595, C2731596 |
| Serotonin Receptor Interactions | C4033645, C4033056, C4033644, C4033055, C4033643, C4033054, C4033642, C4033053, C4033641, C4033052, C4033640, C4033051 |
| Serotonin Transporter Interactions | C0717518, C1655464, C1169992, C0689387, C2073821, C0692744, C0693401, C0707965, C0792947, C0792948, C0978225, C1966345, C1967316, C0689381, C2683249 |
| Serotonin Uptake Inhibitors | C0688670, C0976542, C0976541, C0874810, C0710930, C0355323, C2962549, C2962553, C2962555, C2962557, C2962561, C2962564, C0703031, C0703032, C0703033, C0978918, C1168677, C0688473, C0706344, C0688474, C0706345, C0688475, C0706348, C0688476, C0706350, C0706351, C0688478, C0706353, C3245010, C3249485, C3249491, C3652284, C3651460, C3663526, C3663532, C3663534, C3663536, C3663538, C3663540, C3663543, C3663545, C3663548, C3665175, C3665177, C3667587, C3667588, C3667589, C3667611, C0590946, C3857250, C3857220, C4018221, C4026301, C4048436, C4048203, C4048741, C4048738, C1169470, C1169471, C1595262, C1169801, C4238276, C4238280, C4238282, C4317352, C4317350, C0687887, C0687888, C0687889, C0687890, C0689818, C0689819, C0692697, C0692887, C0692963, C0692964, C2146612, C0693480, C0694411, C0706410, C3709761, C0708673, C0708674, C0709632, C0709644, C0710471, C0711085, C0711098, C0711111, C0711156, C0714037, C0714038, C0714925, C0716299, C0716374, C5207667, C5207500, C5207668, C5207501, C5207669, C5207502, C5207670, C5207503, C5384142, C5384143, C5384144, C5384145, C0791893, C0793206, C5573197, C5679138, C0875775, C0939185, C0976983, C0979061, C0979300, C0980855, C0980857, C0980858, C0980962, C0980963, C0980964, C0980967, C0981443, C0981449, C0981790, C0991723, C1164893, C1168813, C1168851, C1169895, C1276910, C1329174, C1329175, C1329176, C1329177, C1329613, C2928504, C1339840, C1351193, C1585398, C1585399, C1509241, C1656295, C1532660, C1719821, C1720300, C1720061, C1742815, C1949277, C1329935, C1329937, C0716075, C0361135, C2341613, C2341630, C2317356, C2316847, C2369531, C2369534, C2369536, C2369538, C2608681, C2608684, C2608686, C2608688, C2608689, C2608691, C2608692, C2608694, C2608695, C0981532, C0689184, C0689188, C0689187, C0355262, C0689186, C0689185, C0689181, C0874811, C0690610, C0589987, C1306044, C0981208, C0974280, C0687872, C0974281, C0715047, C0981269, C0698280, C0687874, C0687875, C1997479, C0786990, C0542923, C0688370, C0698288, C0688371, C0698289, C0688372, C0707125, C0980147, C0938967, C0787445, C0793359, C2730277, C2730281, C2731196, C2731200, C2731202, C2731204, C2731206, C2740386, C2740390, C2740393, C2740395, C2746487, C2746489, C2746491, C2746493, C2317377, C2316591, C0977007, C0977008, C0981448, C0690210, C0690211, C2747641, C2747645, C2747647, C2747649, C1711760, C1812859, C2316499, C2683197, C2316500, C2609731, C2316502, C2609733, C0874618 |
| Unknown Cellular or Molecular Interaction | C2946966, C2946973, C2946975, C3249370, C3249372, C3472762, C3472794, C3652270, C3651378, C4026033, C4026035, C4026038, C4026039, C4026040, C4026041, C4026042, C4026043, C0717518, C1655464, C1169992, C0689387, C2073821, C0692744, C0693401, C0707965, C5553831, C5553832, C5553833, C5553834, C0978225, C1813811, C1813813, C1813815, C1828654, C1828655, C1828656, C2718476, C2718478, C2718480, C2718482, C2718497, C0692873, C0688071, C0542894, C1169245, C0688072, C0355063, C2723100, C2723102, C0937519 |

| **Table S3b. Concept IDs for Medication Classes under Medications for Diabetes, Antihypertensive, and Lipid-lowering** | |
| --- | --- |
| **Class** | **Concept ID** |
| ACE | C0981290, C0974833, C0974288, C0974287, C1711695, C0974834, C3245003, C0974839, C0974838, C0974837, C3206786, C0709182, C0710686, C1711693, C0715595, C0715559, C3214381, C0709196, C0974836, C1169851, C0709362, C1695067, C1695068, C0974835, C3214383, C0974289, C0709206, C0709231, C1165019, C0709322, C0715607, C0709301, C3234754, C3234752, C0712973, C0693653, C0688115, C0975226, C3210197, C0697926, C3210200, C0713109, C0688114, C0697930, C0697928, C0975229, C0713045, C0975227, C0688116, C0713083, C0991765, C0713069, C0688113, C1177489, C3227017, C0875732, C0976598, C3216969, C0706701, C1712684, C0706700, C0976602, C3206397, C0976597, C0876617, C0976600, C0976595, C0976599, C0706705, C3216974, C3665250, C0874623, C1347373, C1273906, C0976596, C3216971, C0714224, C0706702, C0706703, C0976601, C3241472, C0713686, C0706618, C0876710, C0875882, C0706619, C0981441, C0981433, C0977029, C0977031, C3216996, C0977032, C3216998, C3236067, C0978223, C0707937, C2343862, C1712903, C0707935, C0689378, C3216455, C0707945, C0716558, C0981555, C0709418, C0709416, C0709417, C0354404, C0689379, C0354402, C0981556, C0707946, C0709415, C0705982, C3218961, C0707936, C0689376, C3652804, C0354401, C0689380, C0978224, C0709413, C0709414, C0354403, C0707940, C3232189, C3241025, C3882821, C0875742, C0875741, C3882820, C0979391, C3882727, C0979390, C0876689, C3883662, C3219683, C0979392, C1273362, C3222568, C0981701, C0977529, C0709328, C0709327, C0709330, C1585041, C0875839, C0977516, C0979921, C0875840, C0709332, C1585042, C3212294, C0979920, C0977517, C0875841, C3217101, C0979922, C1585077, C3220233, C0876514, C1964808, C0793972, C1618482, C1161569, C3219075, C0690272, C0690273, C1319665, C0793971, C1617967, C1964807, C1964809, C1740080, C1319670, C0874695, C1964806, C3223373, C2740118, C0692785, C0712592, C0714220, C2740140, C0692784, C0714222, C0714219, C2740145, C0712591, C0692786, C0712590, C3217078, C3217080, C2740117, C0714221 |
| AOM | C2732211, C4293770, C4293738, C3217545, C2732207, C3871482, C3872263, C3857220, C3858229, C3475202, C3475167, C3475169, C3475191, C3475180, C4283096, C4275883, C3474291, C3474286, C3474289, C0732648, C0981643, C1873924, C1949463, C3240167, C3222622 |
| ARB | C3211042, C3211044, C2356110, C2356112, C0716395, C1613105, C0716396, C1657021, C0595602, C0716397, C0705854, C1655297, C1658229, C0939041, C0939042, C1697765, C3226248, C0875788, C0875789, C1169959, C0976694, C1168859, C0976695, C1169960, C3216252, C1644773, C0977513, C0595299, C0595297, C0693959, C0693960, C0875698, C1576497, C0595300, C3216487, C0693958, C3215677, C0875699, C0977511, C3229648, C0875689, C0361503, C3226428, C0707312, C3206788, C2926712, C2926711, C1965884, C1164941, C1169817, C1965443, C1965446, C3217099, C1164978, C1329760, C1966104, C2926717, C1164973, C1169816, C2926702, C2930060, C1329762, C2926707, C1329059, C1965907, C1329761, C2926706, C1965908, C1329060, C3213945, C1965450, C1329058, C2930061, C1965448, C1169818, C2930065, C1965442, C2926716, C3224939, C1509758, C2724493, C0939123, C0705909, C0705908, C2724503, C0937286, C2724508, C3217103, C0939094, C2724512, C3215818, C0716469, C0937285, C0716470, C0936634, C0939095, C2724498, C1585690, C3231974, C4032760, C2722734, C4032803, C1164734, C2684020, C3206792, C2683997, C2684015, C1169614, C1961870, C0693534, C0716230, C1165066, C1949336, C2684016, C1164918, C4032802, C1961869, C3206782, C4032759, C3218288, C2684005, C0694252, C1961871, C0694253, C1164735, C4034138, C3217105, C2684011, C2684010, C2684001, C1949332, C1711675, C2684006, C0693630, C1711677, C1164736, C0593709, C1949334, C1169352, C4032804, C1949335, C2722730, C4032761, C0593708, C2722798, C1176455, C1169897, C0716231, C1961872, C2684000, C1711674, C1169353, C1711672, C4270926, C2722796, C3225788 |
| Biguanides | C0978485, C3265894, C0978482, C3848983, C3264607, C4290578, C4300858, C1876536, C3264980, C1329932, C1168833, C4032972, C2948205, C4290579, C3264620, C0978483, C1628533, C1615632, C4535600, C3859802, C2732708, C4535612, C3264606, C3859753, C1585434, C3264600, C2948201, C1585447, C2948208, C4050749, C3264981, C3264976, C3215942, C1168838, C0977189, C3215934, C4300854, C3859801, C1876537, C1612317, C2733400, C3264614, C2356105, C1169917, C0939008, C3817509, C1329851, C3534941, C4290580, C4050755, C1169919, C2355884, C3848731, C0977192, C1329024, C1165145, C4256513, C3859323, C1168832, C2346234, C4290615, C2364602, C4256619, C3848208, C1168837, C1329852, C3215940, C0939007, C1329120, C3264970, C3220086, C4290577, C4256618, C3534940, C1735400, C3859799, C1168839, C0939052, C2740932, C4290616, C3264613, C4300867, C3215947, C4050757, C4049970, C1329121, C3534995, C3264986, C3215944, C2719785, C3859455, C4256512, C3264984, C3859451, C4300863, C1169925, C0701679, C3848734, C1169924, C4051104, C3859442, C4535596, C3848732, C2948212, C3848209, C4535614, C3848733, C1169918, C3534994, C1628274, C3219462, C1948390, C3848210, C2948219, C1329173, C4535606, C0716527, C1948392, C0939006, C0977187, C1169923, C2948215, C3189634, C0978484, C2356104, C4050761, C3848211, C0701677, C2364606, C2740938, C3225326, C3225336, C3226679, C3239101 |
| GLP1-RA | C3257796, C3218085, C2683919, C2683917, C3257791, C2683912, C2683915, C3257795, C3871482, C3872263, C3817494, C3816985, C3817896, C3817075, C3819902, C3857222, C3857252, C3858337, C3857251, C3857221, C3858233, C4535012, C4521383, C4535014, C4535019, C4535018 |
| Insulin | C1236539, C0977777, C1169459, C0716500, C3226742, C3226735, C1169462, C2683552, C0977786, C0981515, C3226741, C2683543, C2683551, C3226744, C0361079, C0977796, C1949548, C3232043, C0716501, C0977778, C3232042, C2683557, C0977787, C3232044, C0977793, C1949544, C2683547, C2683612, C1169967, C1169537, C3232035, C2683545, C3211885, C3834140, C4478046, C0977811, C0780404, C2683559, C4026351, C0977809, C3211884, C0875843, C0872645, C3888220, C3211883, C2683565, C2683561, C2683601, C2683563, C2683603, C3222730, C2683609, C2356251, C3225371, C4530768, C4056298, C4056297, C2683592, C1693555, C3222105, C1702411, C3229603, C2683584, C0939651, C3893079, C4692193, C4025915, C1532971, C2683590, C3215056, C4293608, C4292822, C4073423, C0977804, C4688827, C4293382, C3211882, C2683582, C0775205, C3211893, C1253297, C0977788, C0785937, C0875569, C2683541, C0795633, C0977790, C2683620, C0977813, C0977818, C2683550, C0977820 |
| Loop | C0706340, C0706341, C3222845, C0706342, C0688058, C0936207, C0688059, C3205871, C0688060, C1245194, C3227234, C0706587, C0697826, C0688816, C0688818, C0709538, C3226475, C0709544, C0709543, C0709536, C0709545, C0690837, C3217073, C0690835, C0690838, C0499011, C0690836, C3217074, C0697300, C0697292, C0706648, C3229626, C0688950, C0977056, C0688946, C0779693, C0688949, C0309152, C4048365, C0992358, C2741653, C1128078, C3217036, C2745885, C0981442, C3193935, C1344834, C3248778, C3217034, C3249807, C1814214 |
| Multi-ingredients | C3536292, C3534941, C3534940, C3534995, C3534994, C4290578, C3848870, C4290579, C4290580, C3848208, C4290577, C3848209, C3848210, C3848211, C3848983, C3848731, C4290615, C4290616, C3848734, C3848732, C3848733, C3859802, C3859753, C3859801, C3817509, C3859323, C3859799, C3859455, C3859451, C3859442, C4300858, C4050749, C4300854, C4050755, C4300867, C4050757, C4049970, C4300863, C4051104, C4050761, C4050753, C4476321, C4535600, C4535612, C4535596, C4535614, C4535606, C1168838, C1168837, C1168839, C1169925, C1169924, C3219462, C1169923, C0977189, C0939008, C0977192, C0939007, C3220086, C0939006, C0977187, C3265894, C3264607, C3264620, C3264606, C3264600, C3264614, C4256513, C4256619, C4256618, C3264613, C4256512, C4275429, C1628533, C1615632, C2732708, C3215934, C1612317, C2733400, C2740932, C1628274, C2740938, C2356105, C2364602, C2356104, C2364606, C1168833, C1169917, C1329851, C1169919, C1165145, C1168832, C1329852, C3215940, C1329120, C1329121, C1169918, C2948205, C2948201, C2948208, C3215942, C2948212, C2948219, C2948215, C1876536, C3264980, C3264981, C3264976, C1876537, C3264970, C3264986, C3215944, C3264984, C1948390, C1948392 |
| Phentermine | C0710708, C0710710, C4283644, C4283642 |
| SGLT-2i | C4535689, C4535693, C4535687, C4535683, C4535600, C4535612, C4535691, C4535706, C4535596, C4535614, C4535606, C3554969, C3556729, C3554970, C3848983, C4290578, C4290579, C3554852, C3556905, C4290580, C3848731, C4290615, C3848208, C4290577, C4290616, C3554853, C3848734, C3848732, C3848209, C3848733, C3848210, C3848211, C3848128, C3848865, C3848127, C4300858, C3848979, C3883463, C4050749, C4300854, C4050755, C3883627, C3882863, C3848729, C4300867, C3812152, C4050757, C3882660, C4049970, C4300863, C4051104, C3882678, C4050761, C3669639, C3669412, C3701137, C3859802, C3859753, C3859801, C3668918, C3817509, C3859323, C3859799, C3709919, C3859455, C3859451, C3859442, C3668919, C4283059 |
| Statins | C1679828, C0593903, C1682654, C1624058, C1624059, C1679830, C1625164, C1624618, C1654635, C0872877, C1680596, C1585149, C1682656, C1623524, C1682328, C0693628, C1678812, C0593905, C0876711, C1624619, C1679348, C0693627, C1624057, C1329216, C3206784, C3212687, C0693848, C1585160, C0593904, C1329218, C1624620, C1625165, C3231323, C0977004, C0939067, C1178718, C0977005, C0353573, C0353572, C3220294, C1615671, C0707313, C0689404, C1612356, C1169849, C1724243, C1177905, C1363119, C0707314, C1613758, C2919995, C1329608, C2919997, C0689406, C3219600, C0710687, C0689405, C2919996, C1612908, C1724748, C1613207, C1169848, C1363118, C1177906, C2726408, C1612652, C3231433, C3224121, C0710619, C3208242, C0710618, C0979724, C1168713, C1169702, C1878303, C1878305, C0979725, C0710617, C1329076, C1329078, C3216026, C0979726, C1878307, C1878306, C3238438, C1319657, C1329652, C1319662, C1319663, C1329651, C1329650, C3212406, C1329114, C1329836, C3227096, C3213147, C1585916, C3244858, C2926692, C0690321, C0980171, C4238194, C3244838, C4275476, C1585921, C0709711, C2241845, C2241847, C0980170, C1509285, C0716341, C2242238, C1585915, C2241335, C1509880, C0354663, C3499546, C3218086, C2242234, C3212521, C1816351, C2926681, C1509287, C0694372, C0981738, C3499539, C2926688, C2926685, C3244852, C1509286, C1509284, C2242230, C3244842, C0354662, C0593254, C1585914, C3241707, C4530572, C2720035, C2720037, C3217589, C2720026, C2720033, C4530573, C2720029, C2720031, C4530574, C3233016 |
| Sulfonylureas | C0688311, C0688310, C0356383, C0698531, C1694131, C0595641, C1813188, C1948933, C1948904, C3211665, C1693552, C3211667, C1948905, C1735669, C1813190, C1630666, C0692629, C0595111, C1695219, C3211663, C1628359, C0692630, C1648661, C0595643, C0692628, C1736208, C0706850, C0706855, C0795264, C0977190, C0977191, C0977193, C0706852, C0977189, C0981456, C0688969, C0939008, C0874626, C0977188, C0706851, C0706859, C0977192, C0939007, C3220086, C0706858, C0779712, C3210639, C0876560, C0874625, C0706857, C0706854, C0939006, C0977187, C0706853, C3224779, C3226000, C0706782, C0706784, C0875801, C3225328, C0706783, C0706781, C0981462, C1168838, C0982738, C0977134, C1168837, C0977136, C3219464, C1168839, C0779711, C0977135, C1169925, C1169924, C3219462, C1169923, C3229854, C0709407, C0709406, C0709405, C0690600, C0690601, C3231998, C0690604 |
| Thiazide Diuretics | C3234796, C0708156, C0708155, C0689201, C0779717, C0689200, C3210738, C1273362, C0714109, C3232003, C1577503, C0709492, C0690640, C2926712, C2926711, C2955630, C0716052, C0717019, C1509758, C2240451, C3211042, C0975000, C0977529, C2240450, C1966492, C0709542, C0977538, C1358771, C0981290, C0716053, C3216469, C0688115, C0709429, C0689378, C2241025, C1827569, C0977534, C0717017, C3210197, C3216455, C0974834, C2684020, C0977535, C0977527, C3216483, C0977499, C0709532, C2356110, C3217099, C0977525, C1165020, C2683997, C0689562, C2684015, C0709390, C0937286, C0689077, C2356112, C0976602, C0782133, C2241021, C1644773, C3216463, C0975002, C0709418, C0709416, C1585041, C0977503, C0716230, C0977500, C1329760, C1244660, C0974837, C0716522, C3217108, C2684016, C0875839, C0709417, C1164918, C0974218, C0778782, C0713109, C2926717, C0689379, C0688470, C3217103, C0977513, C0688114, C0876710, C0709388, C0977516, C0939094, C3206782, C3217107, C2926702, C2930060, C0710686, C1329762, C2684005, C0694252, C0709495, C0689081, C0694253, C0693606, C2955640, C3206127, C0875840, C0875882, C0709394, C2926707, C0981476, C0709415, C1329059, C0713045, C0709397, C3214381, C0688116, C0977528, C1169959, C3217105, C0709535, C1654783, C2684011, C0974836, C2241013, C0975001, C0787090, C2684010, C2684001, C0876617, C0709399, C3212163, C1711675, C0977522, C0709465, C2955638, C3216457, C1585042, C2955635, C0703509, C0717018, C2684006, C0690638, C0977514, C1966491, C0937285, C3216453, C1711677, C0713083, C1329761, C0709362, C0688469, C0712138, C0306756, C1169790, C1827975, C0977532, C2926706, C0981478, C3209169, C0875698, C2240453, C0977517, C0689561, C2240452, C0977533, C1168859, C0689380, C0689079, C0690398, C0977029, C0709537, C2955641, C0709483, C1329060, C0306757, C1628360, C1244658, C0874623, C2241017, C0976596, C1655297, C0690399, C0939095, C1576497, C1169960, C3216971, C3216996, C0977524, C0709413, C1658229, C0875841, C3217101, C1329058, C3216487, C0977032, C0977501, C0939041, C0709501, C0709391, C2955644, C0709414, C1176455, C0709582, C3219142, C0977523, C3215887, C2930061, C0716231, C1585690, C2684000, C0709578, C0875699, C1711674, C3218570, C0977511, C0709593, C0709322, C0689559, C0713069, C1711672, C0977515, C3215885, C0939042, C1585077, C0709301, C0977536, C0688471, C2930065, C0688113, C2926716, C0977518, C3238717, C0717255, C0688315, C0981339, C0717262, C3210228, C0687942, C3249325, C3208842, C0717258, C0975614, C0714249, C0688318, C0697807, C3249324, C0717260, C0717256, C0714317, C0687941, C3210240, C0975613, C3249331, C0688317, C3249318, C0688316, C0708585, C3240792, C0708580, C0708581, C0689598, C0978606, C3217823, C0689597, C0689596, C0708351, C3217191, C0697290, C0706332, C0689550, C3208343, C0716490, C0716513, C0689558, C1531657, C0688253, C0688255, C0975547, C3208341, C0716579, C0716503, C3227205 |
| TZD | C3229645, C0875727, C0875726, C0875725, C1694131, C1168833, C1948933, C1948904, C0980052, C3211665, C1693552, C1169917, C1329851, C1948905, C1169919, C1165145, C1168832, C1329852, C3215940, C1630666, C0980050, C1329120, C0980051, C1695219, C1329121, C1628359, C1648661, C1169918, C0875753, C0875751, C3224064, C0875752, C1813188, C1628533, C1615632, C2732708, C3534938, C3215934, C1612317, C2733400, C3534992, C3534988, C1735669, C3534935, C3534991, C1813190, C3534936, C2740932, C3217546, C0993194, C3534989, C3211663, C0979547, C3534993, C0979548, C3534990, C1628274, C1736208, C3536525, C2740938 |

| **Table S3c. Concept IDs for Medication Classes under Medications for Hormonal Therapy for Menopause** | |
| --- | --- |
| **Class** | **Concept ID** |
| Progesterone | C0028356, C0033308, C0044971, C0068980, C0353462, C0544374, C0698564, C0698565, C0710968, C0773966, C0773969, C0773970, C0773971, C0779772, C0873071, C0874481, C0875809, C0876694, C0979053, C0979793, C0979795, C0979796, C0993195, C1246799, C1329189, C1329190, C1329528, C1586543, C1612541, C1648910, C1816337, C1961967, C2609512, C2757070, C2978012, C2978016, C3189590, C3189633, C3213584, C3214228, C3216616, C3216619, C3218905, C3227100, C3227157, C3230262, C3233877, C3235130, C3236312, C3700931, C3710239, C4083162, C4555372, C4555374 |
| Estrogen Progestin Combination | C0722752, C0779168, C0779169, C0781218, C0781282, C0875685, C0875686, C0973824, C0976869, C0979763, C0994975, C0994979, C1164381, C1249634, C1320116, C1321187, C1321189, C1359783, C1443715, C1443716, C1572362, C1572363, C1828558, C1828578, C1962543, C1968089, C1968460, C1995479, C2243105, C2917166, C2920137, C2979520, C2979524, C3163546, C3211777, C3219430, C3222037, C3222040, C3239047, C3239617, C3466218, C3467505, C3832603, C3833583, C3857806, C3857807, C3871554, C4059003, C4081022, C4081024, C4238122, C4282493, C4282494, C4301303, C4734146, C4740950, C4746047 |
| Estrogen | C0015011, C0305489, C0305490, C0305843, C0305844, C0305845, C0306610, C0306844, C0307026, C0307027, C0307028, C0307030, C0307031, C0307036, C0307039, C0307140, C0307141, C0307142, C0307143, C0307144, C0307145, C0307146, C0307147, C0307906, C0307907, C0307908, C0307909, C0307948, C0307949, C0351023, C0360514, C0360515, C0361215, C0543406, C0544372, C0594669, C0688786, C0688787, C0688788, C0688827, C0688828, C0689808, C0693172, C0698907, C0698954, C0698955, C0701768, C0702471, C0705994, C0706588, C0706785, C0706804, C0706830, C0706893, C0706894, C0707116, C0707117, C0707291, C0707292, C0707465, C0707492, C0707493, C0707494, C0707495, C0707496, C0707497, C0707735, C0707736, C0707737, C0707738, C0707877, C0707878, C0707879, C0707880, C0707881, C0707997, C0707998, C0707999, C0708000, C0708001, C0708002, C0708178, C0710848, C0710849, C0710850, C0710851, C0710852, C0710853, C0710855, C0710856, C0716571, C0717761, C0718341, C0721168, C0732025, C0773954, C0773955, C0773961, C0773978, C0773993, C0777571, C0778505, C0779078, C0779609, C0780174, C0780693, C0780752, C0781218, C0781282, C0781942, C0781943, C0787367, C0787494, C0789699, C0789915, C0789930, C0790502, C0790510, C0791114, C0791720, C0792087, C0792402, C0792403, C0792795, C0792858, C0793327, C0793328, C0872848, C0874641, C0874674, C0875665, C0875668, C0875670, C0875675, C0875680, C0875685, C0875686, C0875806, C0938685, C0938946, C0938983, C0939066, C0939333, C0973824, C0976366, C0976742, C0976743, C0976744, C0976745, C0976746, C0976747, C0976748, C0976749, C0976752, C0976753, C0976754, C0976755, C0976756, C0976757, C0976758, C0976759, C0976760, C0976762, C0976763, C0976764, C0976765, C0976766, C0976767, C0976769, C0976806, C0976807, C0976808, C0976809, C0976810, C0976814, C0976869, C0977095, C0977096, C0978230, C0978469, C0978470, C0978471, C0978915, C0978916, C0979055, C0979149, C0979151, C0979152, C0979160, C0979770, C0979923, C0981629, C0981634, C0981635, C0981637, C0982720, C0982724, C0982725, C0982727, C0982796, C0982797, C0994356, C0994363, C0994365, C0994366, C0994367, C0994424, C0994427, C0994889, C0994982, C1164261, C1164262, C1164381, C1168699, C1168899, C1168948, C1168953, C1168954, C1169592, C1169678, C1177151, C1236561, C1236622, C1237184, C1237185, C1237187, C1237189, C1239557, C1242480, C1244378, C1244828, C1245758, C1246116, C1246117, C1246118, C1246119, C1246120, C1246121, C1246122, C1246124, C1246145, C1246146, C1246147, C1246148, C1246149, C1246160, C1246161, C1246162, C1246362, C1246649, C1247175, C1247177, C1247179, C1247183, C1247184, C1247803, C1248529, C1249634, C1249672, C1249960, C1250402, C1250403, C1252951, C1252964, C1253214, C1254127, C1254148, C1273888, C1273891, C1276728, C1276813, C1276928, C1300016, C1302929, C1302944, C1302958, C1305802, C1320132, C1321178, C1321179, C1321187, C1321188, C1321189, C1321195, C1321676, C1321679, C1329127, C1329128, C1329861, C1329862, C1330622, C1331670, C1359783, C1362988, C1368406, C1368819, C1368835, C1368837, C1368838, C1368839, C1368840, C1368904, C1368937, C1369278, C1373648, C1375652, C1377772, C1378266, C1378411, C1382713, C1383962, C1443629, C1443688, C1443700, C1509421, C1531888, C1563023, C1572276, C1572279, C1572356, C1572625, C1577776, C1584772, C1584773, C1585056, C1585715, C1585862, C1586292, C1586366, C1586367, C1586369, C1586370, C1586371, C1586483, C1589590, C1589606, C1608282, C1608284, C1612313, C1612366, C1615628, C1616584, C1618272, C1618791, C1620908, C1620909, C1628352, C1628355, C1629526, C1629532, C1630663, C1636671, C1654169, C1656811, C1691594, C1691771, C1691955, C1692182, C1692274, C1692840, C1693345, C1694573, C1697782, C1699917, C1712148, C1725783, C1813071, C1813073, C1813075, C1813158, C1814890, C1815659, C1815660, C1815662, C1815663, C1815664, C1815665, C1815668, C1815674, C1815675, C1815676, C1815677, C1815678, C1815679, C1815680, C1815681, C1815682, C1815683, C1815685, C1815687, C1815688, C1815690, C1827854, C1828124, C1828558, C1828578, C1829337, C1875894, C1875895, C1875896, C1876329, C1876330, C1876332, C1876334, C1876335, C1876337, C1876891, C1876892, C1876896, C1876898, C1876905, C1876911, C1877624, C1877639, C1877640, C1877740, C1877741, C1877749, C1877765, C1948371, C1948859, C1949338, C1951555, C1960130, C1960788, C1962427, C1962428, C1962429, C1962431, C1962470, C1962543, C1962572, C1962905, C1962907, C1963689, C1963697, C1964268, C1965186, C1965187, C1965891, C1966660, C1966669, C1968087, C1968088, C1968089, C1968097, C1968100, C1968191, C1968193, C1968276, C1968277, C1968278, C1968279, C1968287, C1968296, C1968297, C1968300, C1968306, C1968307, C1968308, C1968309, C1968354, C1968355, C1968373, C1968383, C1968384, C1968392, C1968393, C1968422, C1968423, C1968424, C1968425, C1968426, C1968427, C1968428, C1968429, C1968430, C1968431, C1968432, C1968460, C1995162, C1995163, C1995165, C1995166, C1995451, C1995479, C1995497, C1995600, C1995670, C1995671, C1995682, C1996296, C1996438, C1996475, C2240404, C2241283, C2241910, C2242038, C2242152, C2242301, C2242307, C2315802, C2340999, C2341479, C2344013, C2344339, C2345890, C2366435, C2366436, C2366553, C2368837, C2608219, C2609577, C2684107, C2684108, C2702035, C2702036, C2702277, C2710363, C2710364, C2726483, C2756661, C2912137, C2912139, C2912140, C2912142, C2912144, C2912145, C2912146, C2917166, C2917854, C2927435, C2937618, C2937622, C2937624, C2937632, C2937636, C2938019, C2939579, C2939581, C2939584, C2939585, C2940276, C2940363, C2940655, C2940656, C2940657, C2949686, C2949687, C2954668, C2955537, C2955538, C2955539, C2963056, C2979087, C2979520, C2979524, C2979772, C2979773, C2980150, C2981104, C3152383, C3152384, C3152386, C3153357, C3153441, C3153562, C3153563, C3153681, C3154691, C3160196, C3160224, C3163546, C3189801, C3189810, C3194708, C3203442, C3205983, C3205984, C3208073, C3208074, C3208075, C3208076, C3208697, C3208698, C3209092, C3211273, C3211274, C3211275, C3211276, C3211277, C3211278, C3211279, C3211627, C3211770, C3211771, C3211772, C3211773, C3211774, C3211775, C3211776, C3211777, C3211778, C3211779, C3211780, C3211781, C3211782, C3211783, C3211784, C3211785, C3211786, C3211787, C3211788, C3211789, C3212732, C3212733, C3212734, C3212735, C3215010, C3215011, C3215012, C3215157, C3215158, C3215358, C3215359, C3215360, C3215361, C3215362, C3215363, C3215605, C3217484, C3217485, C3217486, C3217487, C3218459, C3218460, C3219411, C3219412, C3219413, C3219414, C3219415, C3219416, C3219417, C3219418, C3219419, C3219422, C3219423, C3219424, C3219425, C3219426, C3219427, C3219428, C3219429, C3219430, C3219431, C3219432, C3219433, C3219434, C3219435, C3219436, C3219437, C3219438, C3219440, C3219441, C3219442, C3219443, C3220685, C3220686, C3221184, C3221186, C3221750, C3223056, C3223864, C3223880, C3225753, C3225757, C3226398, C3226399, C3227133, C3227158, C3227159, C3227168, C3234523, C3234524, C3235322, C3236520, C3240886, C3248777, C3249384, C3254884, C3254885, C3254995, C3255000, C3257281, C3257729, C3257730, C3265269, C3267312, C3282692, C3282693, C3465458, C3465720, C3465810, C3465851, C3466118, C3466218, C3474231, C3474397, C3474403, C3474642, C3475376, C3486033, C3486050, C3486073, C3486355, C3496733, C3496734, C3497669, C3497969, C3500781, C3502872, C3502929, C3502954, C3502983, C3503358, C3503397, C3503448, C3503493, C3503556, C3503597, C3503679, C3504926, C3504944, C3504954, C3504956, C3505698, C3530610, C3535409, C3535412, C3535554, C3555414, C3556190, C3556752, C3556753, C3642890, C3643213, C3643647, C3643648, C3644094, C3645016, C3645105, C3645106, C3645320, C3645321, C3665026, C3665027, C3665705, C3666805, C3667137, C3667845, C3667846, C3668515, C3695472, C3696413, C3696712, C3696738, C3696739, C3700577, C3700637, C3817434, C3817658, C3831924, C3832603, C3833742, C3853792, C3856175, C3857712, C3857806, C3857807, C3864705, C3892614, C3892616, C3892694, C4020154, C4026306, C4027248, C4046565, C4046594, C4049792, C4049793, C4049794, C4049795, C4049855, C4049907, C4058358, C4058949, C4059003, C4059094, C4075472, C4081022, C4081024, C4237576, C4255089, C4255090, C4256563, C4256579, C4256640, C4275617, C4276338, C4276343, C4276350, C4281937, C4282493, C4282494, C4283793, C4284408, C4284412, C4290888, C4291081, C4292862, C4293111, C4293117, C4293119, C4294713, C4294714, C4306525, C4317114, C4317215, C4317890, C4318188, C4318394, C4319749, C4319753, C4321327, C4321328, C4321330, C4321331, C4325125, C4325334, C4520883, C4522204, C4522207, C4529460, C4530070, C4530162, C4530997, C4534669, C4538224, C4538225, C4551799, C4551873, C4552041, C4552783, C4553011, C4553012, C4554011, C4554681, C4695042, C4704168, C4704169, C4704170, C4704357, C4708874, C4708875, C4719753, C4719754, C4719755, C4719756, C4719757, C4719758, C4719759, C4719760, C4723386, C4723387, C4723388, C4723394, C4723627, C4734119, C4734146, C4740950, C4741856, C4741859, C4746041, C4746042, C4746043, C4746123, C4750478, C4756029, C4756077, C4756212, C4756213, C4756309, C4756310, C4756314, C4756315, C4756317, C4756334, C4756335, C4756336, C4756337, C4756339, C4756340, C4756341, C4756342, C4756343, C4756952, C4759111, C4759114, C4759117, C4759120, C4759123, C4759175, C5191527, C5191639, C5191991, C5192155, C5210111, C5210310, C5210311, C5221741, C5234976, C5235017, C5241283, C5241639, C5384264, C5384337, C5384550, C5384573, C5384574, C5398358, C5398438, C5399575, C5401996, C5402298, C5402299, C5402300, C5402306, C5417244, C5417245, C5417246, C5417247, C5417248, C5421812, C5421849, C5424006, C5432403, C5432756, C5439180, C5443520, C5443864, C5449295, C5449296, C5449297, C5455330, C5455332, C5455333, C5455334, C5455335, C5550103, C5575087, C5686351, C5698578, C0590022, C0688794, C0688797, C0688798, C0688800, C0688801, C0688802, C0688807, C0698961, C0698962, C0704314, C0704371, C0710100, C0710590, C0710971, C0710972, C0712544, C0712691, C0714384, C0773963, C0778293, C0779168, C0779169, C0875621, C0875701, C0875702, C0875867, C0976772, C0976773, C0976774, C0976775, C0976776, C0976778, C0976780, C0976781, C0976782, C0976783, C0979763, C0982722, C0994975, C0994979, C1168635, C1168784, C1169850, C1246129, C1253120, C1253147, C1253195, C1253196, C1253197, C1253210, C1253213, C1253277, C1253278, C1253279, C1253286, C1273890, C1302943, C1302954, C1306313, C1329054, C1329755, C1368855, C1368857, C1368858, C1443715, C1443716, C1509419, C1572362, C1572363, C1585048, C1693735, C1693736, C1694326, C1695406, C1697873, C1697874, C1697875, C1699550, C1702914, C1829313, C1964988, C1965090, C1995363, C1995364, C2369748, C2369749, C2930199, C2930206, C2930210, C3216829, C3216830, C3216832, C3216833, C3220018, C3220019, C3220020, C3220021, C3220022, C3220023, C3220024, C3220025, C3220026, C3220027, C3220028, C3220030, C3220031, C3220032, C3220033, C3220034, C3220035, C3220036, C3667262, C3667360, C3668762, C3668763, C3668764, C4074709, C4708475, C4755564, C4755996, C4755997, C4755998, C4755999, C4756000, C5192147, C5548330, C5548331, C5548334, C5566962, C5566963, C0706421, C0706422, C0717211, C0717212, C0717222, C0717243, C0976790, C0976791, C0976792, C0976793, C0976794, C1246132, C1252129, C1368862, C2317505, C2317564, C3216834, C3216835, C3216836, C3216837, C3216838, C5191247, C5191248, C3537515, C3537516, C3537517, C3537518, C3537525 |

| **Table S4. Pearson Correlation Coefficients Among Continuous Must-Include Covariates (using 3-Year cohort, N = 115,942 )** | | |
| --- | --- | --- |
| **Variables** | **Correlation (r)** | **Interpretation** |
| BMI 3 Years Prior & Age | -0.16 | Small negative correlation |
| BMI 3 Years Prior & Elixhauser Comorbidity Score | 0.04 | Essentially no correlation |
| BMI 3 Years Prior & Charlson Comorbidity Score | -0.003 | No correlation |
| Age & Elixhauser Comorbidity Score | 0.48 | Moderate positive correlation |
| Age & Charlson Comorbidity Score | 0.37 | Moderate positive correlation |
| Elixhauser Comorbidity Score & Charlson Comorbidity Score | 0.70 | Strong correlation, expected because both measure comorbidities. |

| **Table S5. Odds Ratio for Primary Cancer Endpoints per 1% Weight Loss** | | | | | |
| --- | --- | --- | --- | --- | --- |
| **Cancer** | **Year Cohort** | **Estimate** | **Corrected p-value** | **Uncorrected p-value** | **Additional Covariates** |
| Colon | 3 years | 0.99 (0.979, 1.001) | 0.094 | 0.08 | Essential hypertension, Migraine / tension / cluster headaches, Musculoskeletal pain, NAFLD/NASH, Sciatica and low back pain, ARB, Biguanides (Metformin class), GABA-A modulators, Hormone receptor agonists, Loop diuretics, SGLT-2 inhibitors |
| Colon | 5 years | 0.99 (0.977, 1.003) | 0.15 | 0.14 | Musculoskeletal pain, NAFLD/NASH, GABA-A modulators, Hormone receptor agonists, Loop diuretics, SGLT-2 inhibitors |
| Corpus Uteri (endometrium) | 3 years | 0.978 (0.966, 0.991) | 0.001 | < 0.001 | Acute myocardial infarction, Cardiovascular disease, Cerebrovascular disease, Gastroesophageal reflux disease, Heart failure, Migraine / tension / cluster headaches, Musculoskeletal pain, Estrogen, Hormone receptor agonists |
| Corpus Uteri (endometrium) | 5 years | 0.974 (0.962, 0.987) | < 0.001 | < 0.001 | Type 2 Diabetes status, Acute stroke, Cardiovascular disease, Gastroesophageal reflux disease, Heart failure, Migraine / tension / cluster headaches, Musculoskeletal pain, Histamine H1 receptor antagonists, Hormone receptor agonists, SGLT-2 inhibitors |
| Corpus Uteri (endometrium) | 10 years | 0.973 (0.956, 0.99) | 0.005 | 0.003 | Type 2 Diabetes status |
| Esophageal adenocarcinoma | 3 years | 0.976 (0.959, 0.994) | 0.012 | 0.008 | Chronic pancreatitis, Musculoskeletal pain, Hormone receptor agonists, Multi-ingredient medications, Serotonin 5-HT3 antagonists |
| Gallbladder | 5 years | 0.97 (0.923, 1.02) | 0.53 | 0.24 |  |
| Liver | 3 years | 0.984 (0.968, 1) | 0.091 | 0.057 | Type 2 Diabetes status, Cerebrovascular disease, Chronic pancreatitis, Heart failure, GLP-1 receptor agonists, Hormone receptor agonists, Loop diuretics |
| Liver | 5 years | 0.987 (0.968, 1.007) | 0.29 | 0.22 | Type 2 Diabetes status, Cardiovascular disease, Cerebrovascular disease, Heart failure, Musculoskeletal pain, Psoriasis, Loop diuretics |
| Liver | 10 years | 0.982 (0.962, 1.002) | 0.17 | 0.078 | Chronic pancreatitis, Loop diuretics |
| Multiple myeloma | 5 years | 0.987 (0.97, 1.005) | 0.17 | 0.16 | Cerebrovascular disease, Heart failure, Musculoskeletal pain, Sciatica and low back pain, Estrogen-progestin combination, Thiazide diuretics |
| Multiple myeloma | 10 years | 0.969 (0.95, 0.987) | 0.004 | 0.001 | GABA-A modulators |
| Overall obesity-related cancer | 3 years | 0.99 (0.984, 0.996) | < 0.001 | < 0.001 | Type 2 Diabetes status, Cardiovascular disease, Cerebrovascular disease, Chronic pancreatitis, Chronic kidney disease / ESRD, Essential hypertension, Heart failure, Migraine / tension / cluster headaches, Musculoskeletal pain, NAFLD/NASH, Estrogen, GABA-A modulators, GLP-1 receptor agonists, Hormone receptor agonists, Loop diuretics, SGLT-2 inhibitors, Thiazolidinediones (TZD) |
| Overall obesity-related cancer | 5 years | 0.989 (0.984, 0.995) | < 0.001 | < 0.001 | Type 2 Diabetes status, Cardiovascular disease, Cerebrovascular disease, Chronic pancreatitis, Chronic kidney disease / ESRD, Essential hypertension, Heart failure, Musculoskeletal pain, ACE inhibitors, Adrenergic alpha antagonists, ARB, Estrogen, Estrogen-progestin combination, GABA-A modulators, GLP-1 receptor agonists, Hormone receptor agonists, Loop diuretics, SGLT-2 inhibitors, Thiazolidinediones (TZD) |
| Overall obesity-related cancer | 10 years | 0.992 (0.984, 1) | 0.057 | 0.052 | Type 2 Diabetes status, Cardiovascular disease, Cerebrovascular disease, Chronic pancreatitis, Chronic kidney disease / ESRD, Heart failure, Musculoskeletal pain, Adrenergic alpha antagonists, Dopamine antagonists, GABA-A modulators, GLP-1 receptor agonists, Loop diuretics |
| Pancreas | 10 years | 0.999 (0.971, 1.028) | 0.97 | 0.96 | Chronic pancreatitis, Heart failure |
| Postmenopausal breast cancer | 3 years | 0.987 (0.973, 1) | 0.06 | 0.054 | Type 2 Diabetes status, Acute kidney failure, Acute myocardial infarction, Cardiovascular disease, Chronic kidney disease / ESRD, Heart failure, Musculoskeletal pain, NAFLD/NASH, Hormone receptor agonists, Insulin, SGLT-2 inhibitors |
| Postmenopausal breast cancer | 5 years | 0.987 (0.973, 1) | 0.066 | 0.056 | Acute kidney failure, Acute myocardial infarction, Cardiovascular disease, Chronic kidney disease / ESRD, Heart failure, Musculoskeletal pain, NAFLD/NASH, Adrenergic alpha antagonists, GABA-A modulators, Hormone receptor agonists, Insulin |
| Postmenopausal breast cancer | 10 years | 0.991 (0.971, 1.011) | 0.46 | 0.39 | Acute kidney failure, Heart failure, Musculoskeletal pain, Pancreatitis, Insulin |
| Rectum | 3 years | 0.994 (0.974, 1.013) | 0.52 | 0.52 | Essential hypertension, Heart failure, Osteoarthritis, Sciatica and low back pain, GABA-A modulators, GLP-1 receptor agonists |
| Rectum | 5 years | 0.98 (0.963, 0.998) | 0.057 | 0.026 | Heart failure, Osteoarthritis, Estrogen, Serotonin agonists |
| Renal Cell carcinoma | 3 years | 0.983 (0.971, 0.996) | 0.022 | 0.012 | Type 2 Diabetes status, Musculoskeletal pain, Anti-obesity medications, Insulin |
| Renal Cell carcinoma | 5 years | 0.983 (0.97, 0.997) | 0.026 | 0.015 | Type 2 Diabetes status, Musculoskeletal pain, Insulin |
| Corrected p-values were calculated using false discovery rate (FDR) adjustment and represent the primary basis for the statistical inference. Uncorrected p-values are reported solely for transparency and descriptive purposes; no conclusion should be drawn from them. | | | | | |

| **Table S6. Odds Ratio for Secondary Cancer Endpoints per 1% Weight Loss** | | | | | |
| --- | --- | --- | --- | --- | --- |
| **Cancer** | **Year Cohort** | **Estimate** | **Corrected p-value** | **Uncorrected p-value** | **Additional Covariates** |
| Malignant neoplasm of breast | 10 years | 0.997 (0.984, 1.01) | 0.69 | 0.69 | Type 2 Diabetes status, Musculoskeletal pain, Hormone receptor agonists, Insulin |
| Malignant neoplasms of digestive organs | 3 years | 0.985 (0.977, 0.994) | < 0.001 | < 0.001 | Type 2 Diabetes status, Acute kidney failure, Cardiovascular disease, Cerebrovascular disease, Chronic pancreatitis, Chronic kidney disease / ESRD, Essential hypertension, Heart failure, Migraine / tension / cluster headaches, Musculoskeletal pain, ARB, Estrogen, GABA-A modulators, GLP-1 receptor agonists, Hormone receptor agonists, Loop diuretics, SGLT-2 inhibitors |
| Malignant neoplasms of digestive organs | 5 years | 0.986 (0.977, 0.995) | 0.003 | 0.002 | Cardiovascular disease, Cerebrovascular disease, Chronic pancreatitis, Chronic kidney disease / ESRD, Heart failure, Musculoskeletal pain, ACE inhibitors, ARB, Estrogen, GABA-A modulators, Hormone receptor agonists, SGLT-2 inhibitors |
| Malignant neoplasms of digestive organs | 10 years | 0.991 (0.98, 1.003) | 0.17 | 0.14 | Acute myocardial infarction, Cardiovascular disease, Cerebrovascular disease, Chronic pancreatitis, Chronic kidney disease / ESRD, Heart failure, Musculoskeletal pain, NAFLD/NASH, GABA-A modulators, GLP-1 receptor agonists |
| Malignant neoplasms of eye, brain and other parts of central nervous system | 3 years | 0.976 (0.961, 0.992) | 0.01 | 0.003 | Musculoskeletal pain, GABA-A modulators, SGLT-2 inhibitors |
| Malignant neoplasms of eye, brain and other parts of central nervous system | 5 years | 0.983 (0.966, 1) | 0.13 | 0.047 | Musculoskeletal pain, GABA-A modulators |
| Malignant neoplasms of eye, brain and other parts of central nervous system | 10 years | 0.983 (0.963, 1.003) | 0.23 | 0.094 |  |
| Malignant neoplasms of female genital organs | 3 years | 0.989 (0.98, 0.999) | 0.033 | 0.03 | Cerebrovascular disease, Gastroesophageal reflux disease, Musculoskeletal pain, Estrogen, GLP-1 receptor agonists, Hormone receptor agonists |
| Malignant neoplasms of female genital organs | 5 years | 0.975 (0.966, 0.985) | < 0.001 | < 0.001 | Type 2 Diabetes status, Migraine / tension / cluster headaches, Musculoskeletal pain, Adrenergic alpha antagonists, Adrenergic alpha-1 antagonists, Dopamine antagonists, Estrogen, GLP-1 receptor agonists, Histamine H1 receptor antagonists, Hormone receptor agonists, Serotonin 5-HT3 antagonists |
| Malignant neoplasms of female genital organs | 10 years | 0.986 (0.971, 1.001) | 0.14 | 0.076 | Type 2 Diabetes status |
| Malignant neoplasms of ill-defined, secondary and unspecified sites | 3 years | 0.997 (0.99, 1.004) | 0.45 | 0.43 | Type 2 Diabetes status, Acute myocardial infarction, Cardiovascular disease, Cerebrovascular disease, Chronic pancreatitis, Chronic kidney disease / ESRD, Depression, Heart failure, Musculoskeletal pain, NAFLD/NASH, Estrogen, GABA-A modulators, GLP-1 receptor agonists, Hormone receptor agonists, Loop diuretics, Progesterone, SGLT-2 inhibitors, Thiazolidinediones (TZD) |
| Malignant neoplasms of ill-defined, secondary and unspecified sites | 5 years | 0.998 (0.991, 1.005) | 0.62 | 0.59 | Acute myocardial infarction, Cardiovascular disease, Chronic pancreatitis, Chronic kidney disease / ESRD, Heart failure, Musculoskeletal pain, NAFLD/NASH, ACE inhibitors, GABA-A modulators, GLP-1 receptor agonists, Hormone receptor agonists, Loop diuretics, SGLT-2 inhibitors, Thiazolidinediones (TZD) |
| Malignant neoplasms of ill-defined, secondary and unspecified sites | 10 years | 0.993 (0.983, 1.003) | 0.21 | 0.16 | Type 2 Diabetes status, Acute myocardial infarction, Chronic pancreatitis, Chronic kidney disease / ESRD, Essential hypertension, Heart failure, Musculoskeletal pain, GABA-A modulators, Loop diuretics, SGLT-2 inhibitors |
| Malignant neoplasms of lip, oral cavity and pharynx | 3 years | 0.987 (0.962, 1.013) | 0.43 | 0.31 | Musculoskeletal pain, GABA-A modulators |
| Malignant neoplasms of male genital organs | 3 years | 0.984 (0.975, 0.994) | 0.002 | 0.001 | Acute kidney failure, Acute myocardial infarction, Cardiovascular disease, Essential hypertension, Heart failure, Musculoskeletal pain, NAFLD/NASH, Sciatica and low back pain, Adrenergic alpha antagonists, Adrenergic alpha-1 antagonists, Dopamine antagonists, Hormone receptor agonists, Insulin, SGLT-2 inhibitors, Statins, Thiazolidinediones (TZD) |
| Malignant neoplasms of male genital organs | 5 years | 0.984 (0.975, 0.993) | < 0.001 | < 0.001 | Acute myocardial infarction, Chronic kidney disease / ESRD, Musculoskeletal pain, NAFLD/NASH, Hormone receptor agonists, Loop diuretics, SGLT-2 inhibitors, Thiazolidinediones (TZD) |
| Malignant neoplasms of male genital organs | 10 years | 0.984 (0.971, 0.996) | 0.016 | 0.013 | Acute myocardial infarction, Musculoskeletal pain, NAFLD/NASH, Hormone receptor agonists, Serotonin agonists, Unknown cellular/molecular interaction |
| Malignant neoplasms of mesothelial and soft tissue | 3 years | 0.981 (0.962, 1) | 0.05 | 0.029 | GABA-A modulators, GLP-1 receptor agonists, Thiazolidinediones (TZD) |
| Malignant neoplasms of mesothelial and soft tissue | 5 years | 0.975 (0.959, 0.992) | 0.011 | 0.003 | Loop diuretics |
| Malignant neoplasms of mesothelial and soft tissue | 10 years | 0.975 (0.951, 1) | 0.069 | 0.023 |  |
| Malignant neoplasms of respiratory and intrathoracic organs | 3 years | 0.991 (0.983, 0.998) | 0.017 | 0.013 | Type 2 Diabetes status, Acute kidney failure, Essential hypertension, Musculoskeletal pain, NAFLD/NASH, Pancreatitis, GABA-A modulators, GLP-1 receptor agonists, Hormone receptor agonists, Loop diuretics, SGLT-2 inhibitors, Statins, Thiazide diuretics |
| Malignant neoplasms of respiratory and intrathoracic organs | 5 years | 0.991 (0.984, 0.999) | 0.04 | 0.032 | Type 2 Diabetes status, Cerebrovascular disease, Chronic kidney disease / ESRD, Musculoskeletal pain, NAFLD/NASH, GABA-A modulators, GLP-1 receptor agonists, Hormone receptor agonists, Loop diuretics, SGLT-2 inhibitors, Statins, Thiazide diuretics, Thiazolidinediones (TZD) |
| Malignant neoplasms of respiratory and intrathoracic organs | 10 years | 0.988 (0.978, 0.997) | 0.018 | 0.009 | Musculoskeletal pain, Psoriasis, Sciatica and low back pain, GABA-A modulators, GLP-1 receptor agonists, Insulin, SGLT-2 inhibitors, Thiazolidinediones (TZD) |
| Malignant neoplasms of urinary tract | 3 years | 0.978 (0.969, 0.988) | < 0.001 | < 0.001 | Type 2 Diabetes status, Acute myocardial infarction, Cardiovascular disease, Cerebrovascular disease, Chronic kidney disease / ESRD, Heart failure, Musculoskeletal pain, NAFLD/NASH, GABA-A modulators, Hormone receptor agonists, SGLT-2 inhibitors, Sulfonylureas |
| Malignant neoplasms of urinary tract | 5 years | 0.985 (0.975, 0.995) | 0.003 | 0.002 | Acute myocardial infarction, Cardiovascular disease, Cerebrovascular disease, Chronic kidney disease / ESRD, Heart failure, Musculoskeletal pain, Biguanides (Metformin class), GABA-A modulators, Hormone receptor agonists, Loop diuretics, SGLT-2 inhibitors, Sulfonylureas |
| Malignant neoplasms of urinary tract | 10 years | 0.984 (0.971, 0.998) | 0.047 | 0.027 | Heart failure, Musculoskeletal pain, GABA-A modulators |
| Malignant neoplasms, stated or presumed to be primary, of lymphoid, hematopoietic and related tissue | 5 years | 0.993 (0.984, 1.003) | 0.17 | 0.16 | Cardiovascular disease, Cerebrovascular disease, Chronic kidney disease / ESRD, Heart failure, Musculoskeletal pain, GABA-A modulators, GLP-1 receptor agonists, Hormone receptor agonists, Thiazolidinediones (TZD) |
| Malignant neoplasms, stated or presumed to be primary, of lymphoid, hematopoietic and related tissue | 10 years | 0.986 (0.975, 0.997) | 0.018 | 0.012 | Acute stroke, Migraine / tension / cluster headaches, GABA-A modulators, GLP-1 receptor agonists, Monoamine oxidase inhibitors, Thiazolidinediones (TZD) |
| Melanoma and other malignant neoplasms of skin | 3 years | 0.983 (0.977, 0.99) | < 0.001 | < 0.001 | Type 2 Diabetes status, Acute kidney failure, Acute myocardial infarction, Cerebrovascular disease, Chronic kidney disease / ESRD, Migraine / tension / cluster headaches, Musculoskeletal pain, NAFLD/NASH, Psoriasis, Sciatica and low back pain, Adrenergic alpha-1 antagonists, Dopamine uptake inhibitors, Estrogen-progestin combination, GLP-1 receptor agonists, Hormone receptor agonists, Insulin, Progesterone, Serotonin 5-HT3 antagonists, Serotonin receptor interactions, SGLT-2 inhibitors, Statins, Thiazide diuretics, Thiazolidinediones (TZD) |
| Melanoma and other malignant neoplasms of skin | 5 years | 0.985 (0.979, 0.992) | < 0.001 | < 0.001 | Type 2 Diabetes status, Acute kidney failure, Cerebrovascular disease, Chronic kidney disease / ESRD, Essential hypertension, Musculoskeletal pain, NAFLD/NASH, Estrogen, GABA-A modulators, GLP-1 receptor agonists, Hormone receptor agonists, Insulin, Progesterone, Serotonin 5-HT3 antagonists, SGLT-2 inhibitors, Statins, Thiazide diuretics, Thiazolidinediones (TZD) |
| Melanoma and other malignant neoplasms of skin | 10 years | 0.988 (0.978, 0.998) | 0.019 | 0.016 | Acute kidney failure, Cardiovascular disease, Musculoskeletal pain, Sciatica and low back pain, Adrenergic alpha antagonists, Adrenergic alpha-1 antagonists, Dopamine antagonists, Estrogen-progestin combination, GABA-A modulators, GLP-1 receptor agonists, Histamine H1 receptor antagonists, Hormone receptor agonists, Loop diuretics, Serotonin 5-HT3 antagonists, Serotonin agonists, SGLT-2 inhibitors, Statins, Thiazide diuretics, Thiazolidinediones (TZD), Unknown cellular/molecular interaction |
| Neuroendocrine tumors | 3 years | 0.987 (0.973, 1.002) | 0.20 | 0.08 | Chronic pancreatitis |
| Neuroendocrine tumors | 10 years | 0.988 (0.969, 1.007) | 0.34 | 0.20 | Chronic pancreatitis, Gastroesophageal reflux disease |
| Overall malignant cancer | 3 years | 0.992 (0.989, 0.996) | < 0.001 | < 0.001 | Type 2 Diabetes status, Acute kidney failure, Acute stroke, Cardiovascular disease, Chronic pancreatitis, Chronic kidney disease / ESRD, Depression, Essential hypertension, Heart failure, Migraine / tension / cluster headaches, Musculoskeletal pain, NAFLD/NASH, Sciatica and low back pain, Anti-obesity medications, Biguanides (Metformin class), GABA-A modulators, GLP-1 receptor agonists, Histamine H1 receptor antagonists, Hormone receptor agonists, Insulin, Loop diuretics, Phentermine, Serotonin uptake inhibitors, SGLT-2 inhibitors, Sulfonylureas, Thiazide diuretics, Thiazolidinediones (TZD) |
| Overall malignant cancer | 5 years | 0.994 (0.991, 0.997) | < 0.001 | < 0.001 | Type 2 Diabetes status, Acute kidney failure, Acute myocardial infarction, Acute stroke, Cardiovascular disease, Chronic pancreatitis, Chronic kidney disease / ESRD, Depression, Essential hypertension, Heart failure, Musculoskeletal pain, NAFLD/NASH, Sciatica and low back pain, ACE inhibitors, Anti-obesity medications, Biguanides (Metformin class), Estrogen-progestin combination, GABA-A modulators, GLP-1 receptor agonists, Hormone receptor agonists, Loop diuretics, Serotonin uptake inhibitors, SGLT-2 inhibitors, Statins, Sulfonylureas, Thiazide diuretics, Thiazolidinediones (TZD) |
| Overall malignant cancer | 10 years | 0.991 (0.987, 0.995) | < 0.001 | < 0.001 | Type 2 Diabetes status, Acute myocardial infarction, Acute stroke, Cardiovascular disease, Chronic pancreatitis, Chronic kidney disease / ESRD, Eating disorders, Essential hypertension, Heart failure, Musculoskeletal pain, Sciatica and low back pain, Biguanides (Metformin class), GABA-A modulators, GLP-1 receptor agonists, Loop diuretics, Serotonin receptor interactions, SGLT-2 inhibitors, Sulfonylureas, Thiazolidinediones (TZD) |
| Corrected p-values were calculated using false discovery rate (FDR) adjustment and represent the primary basis for the statistical inference. Uncorrected p-values are reported solely for transparency and descriptive purposes; no conclusion should be drawn from them. | | | | | |

| **Table S7. Subgroup Analyses on Charlson Comorbidity Index ≥ 3 & < 3 (Overall obesity-related cancer & Overall malignant cancer)** | | | | | | | | | |
| --- | --- | --- | --- | --- | --- | --- | --- | --- | --- |
| **Cancer** | **Year Cohort** | | **Subgroup** | | **Estimate** | **Corrected p-value** | **Uncorrected p-value** | **N Case/N Control** | **Additional Covariates** |
| Overall obesity-related cancer | 3 years | CCI ≥ 3 | | 0.989 (0.965,1.013) | | 0.40 | 0.37 | 202/4713 | Acute myocardial infarction, Cardiovascular disease, Cerebrovascular disease, Chronic pancreatitis, Chronic kidney disease / ESRD, Heart failure, NAFLD/NASH, Hormone receptor agonists |
| Overall obesity-related cancer | 3 years | CCI < 3 | | 0.99 (0.983,0.998) | | 0.012 | 0.01 | 1764/108902 | Type 2 Diabetes status, Cardiovascular disease, Chronic pancreatitis, Essential hypertension, Heart failure, Migraine / tension / cluster headaches, Musculoskeletal pain, NAFLD/NASH, Adrenergic alpha antagonists, Estrogen, GABA-A modulators, GLP-1 receptor agonists, Hormone receptor agonists, Loop diuretics, SGLT-2 inhibitors, Thiazolidinediones (TZD) |
| Overall obesity-related cancer | 5 years | CCI ≥ 3 | | 1.004 (0.98,1.028) | | 0.80 | 0.74 | 159/4317 | Acute myocardial infarction, Cardiovascular disease, Chronic pancreatitis, Chronic kidney disease / ESRD, Heart failure, NAFLD/NASH, Hormone receptor agonists |
| Overall obesity-related cancer | 5 years | CCI < 3 | | 0.988 (0.981,0.996) | | 0.003 | 0.002 | 1328/97908 | Type 2 Diabetes status, Cardiovascular disease, Chronic pancreatitis, Essential hypertension, Heart failure, Musculoskeletal pain, ACE inhibitors, Adrenergic alpha antagonists, ARB, Estrogen, Estrogen-progestin combination, GABA-A modulators, GLP-1 receptor agonists, Hormone receptor agonists, Loop diuretics, SGLT-2 inhibitors |
| Overall obesity-related cancer | 10 years | CCI ≥ 3 | | 1.014 (0.984,1.045) | | 0.42 | 0.37 | 59/2536 | Acute myocardial infarction, Cerebrovascular disease, Chronic pancreatitis, Gastroesophageal reflux disease, Heart failure, Migraine / tension / cluster headaches, Hormone receptor agonists, Norepinephrine uptake inhibitors |
| Overall obesity-related cancer | 10 years | CCI < 3 | | 0.992 (0.982,1.002) | | 0.14 | 0.13 | 484/55369 | Type 2 Diabetes status, Cardiovascular disease, Chronic pancreatitis, Essential hypertension, Heart failure, Musculoskeletal pain, Adrenergic alpha antagonists, Dopamine antagonists, GABA-A modulators, GLP-1 receptor agonists |
| Overall malignant cancer | 3 years | CCI ≥ 3 | | 0.98 (0.968,0.993) | | 0.004 | 0.003 | 875/3588 | Acute kidney failure, Acute myocardial infarction, Cardiovascular disease, Cerebrovascular disease, Chronic pancreatitis, Chronic kidney disease / ESRD, Heart failure, Musculoskeletal pain, NAFLD/NASH, Psoriasis, Hormone receptor agonists, Loop diuretics, SGLT-2 inhibitors, Statins, Thiazolidinediones (TZD) |
| Overall malignant cancer | 3 years | CCI < 3 | | 0.992 (0.988,0.996) | | < 0.001 | < 0.001 | 7693/95301 | Type 2 Diabetes status, Cardiovascular disease, Cerebrovascular disease, Chronic pancreatitis, Chronic kidney disease / ESRD, Depression, Essential hypertension, Heart failure, Migraine / tension / cluster headaches, Musculoskeletal pain, NAFLD/NASH, Pancreatitis, Sciatica and low back pain, Anti-obesity medications, Biguanides (Metformin class), Estrogen-progestin combination, GABA-A modulators, GLP-1 receptor agonists, Hormone receptor agonists, Loop diuretics, Serotonin uptake inhibitors, SGLT-2 inhibitors, Sulfonylureas, Thiazide diuretics, Thiazolidinediones (TZD) |
| Overall malignant cancer | 5 years | CCI ≥ 3 | | 0.986 (0.973,0.999) | | 0.057 | 0.04 | 658/3274 | Acute kidney failure, Acute myocardial infarction, Acute stroke, Cardiovascular disease, Cerebrovascular disease, Chronic pancreatitis, Chronic kidney disease / ESRD, Heart failure, Musculoskeletal pain, NAFLD/NASH, Psoriasis, Hormone receptor agonists, Loop diuretics, SGLT-2 inhibitors, Thiazolidinediones (TZD) |
| Overall malignant cancer | 5 years | CCI < 3 | | 0.992 (0.988,0.996) | | < 0.001 | < 0.001 | 5825/86814 | Type 2 Diabetes status, Acute stroke, Cardiovascular disease, Chronic pancreatitis, Chronic kidney disease / ESRD, Depression, Essential hypertension, Heart failure, Migraine / tension / cluster headaches, Musculoskeletal pain, NAFLD/NASH, Sciatica and low back pain, ACE inhibitors, Adrenergic alpha-1 antagonists, Biguanides (Metformin class), Dopamine agonists, Estrogen-progestin combination, GABA-A modulators, GLP-1 receptor agonists, Hormone receptor agonists, Loop diuretics, Serotonin uptake inhibitors, SGLT-2 inhibitors, Statins, Sulfonylureas, Thiazide diuretics, Thiazolidinediones (TZD) |
| Overall malignant cancer | 10 years | CCI ≥ 3 | | 0.992 (0.975,1.01) | | 0.44 | 0.39 | 276/1848 | Acute myocardial infarction, Cardiovascular disease, Cerebrovascular disease, Chronic pancreatitis, Heart failure, NAFLD/NASH, Psoriasis, Sciatica and low back pain, Hormone receptor agonists, SGLT-2 inhibitors |
| Overall malignant cancer | 10 years | CCI < 3 | | 0.991 (0.986,0.996) | | < 0.001 | < 0.001 | 2232/48176 | Type 2 Diabetes status, Acute stroke, Cardiovascular disease, Chronic pancreatitis, Chronic kidney disease / ESRD, Eating disorders, Essential hypertension, Heart failure, Musculoskeletal pain, Biguanides (Metformin class), GABA-A modulators, GLP-1 receptor agonists, Serotonin receptor interactions, SGLT-2 inhibitors, Sulfonylureas, Thiazide diuretics, Thiazolidinediones (TZD) |

Corrected p-values were calculated using false discovery rate (FDR) adjustment and represent the primary basis for the statistical inference. Uncorrected p-values are reported solely for transparency and descriptive purposes; no conclusion should be drawn from them.

| **Table S8. Subgroup Analyses by Gender (Female vs Male) for Overall Obesity-Related and Overall Malignant Cancer** | | | | | | | |
| --- | --- | --- | --- | --- | --- | --- | --- |
| **Cancer** | **Year Cohort** | **Subgroup** | **Estimate** | **Corrected p-value** | **Uncorrected p-value** | **N Case/N Control** | **Additional Covariates** |
| Overall obesity-related cancer | 3 years | female | 0.991 (0.982,0.999) | 0.044 | 0.036 | 1120/59080 | Type 2 Diabetes status, Acute kidney failure, Cardiovascular disease, Cerebrovascular disease, Chronic pancreatitis, Heart failure, Musculoskeletal pain, Sciatica and low back pain, Adrenergic alpha antagonists, Estrogen, GABA-A modulators, GLP-1 receptor agonists, Hormone receptor agonists, SGLT-2 inhibitors, Thiazolidinediones (TZD) |
| Overall obesity-related cancer | 3 years | male | 0.983 (0.971,0.995) | 0.007 | 0.005 | 846/54535 | Type 2 Diabetes status, Cardiovascular disease, Chronic pancreatitis, Chronic kidney disease / ESRD, Essential hypertension, Heart failure, Migraine / tension / cluster headaches, Musculoskeletal pain, Osteoarthritis, GABA-A modulators, GLP-1 receptor agonists, Hormone receptor agonists, Loop diuretics, Serotonin agonists, SGLT-2 inhibitors, Statins, Unknown cellular/molecular interaction |
| Overall obesity-related cancer | 5 years | female | 0.988 (0.979,0.996) | 0.01 | 0.006 | 839/52790 | Type 2 Diabetes status, Acute kidney failure, Cardiovascular disease, Cerebrovascular disease, Chronic pancreatitis, Heart failure, Musculoskeletal pain, Adrenergic alpha antagonists, Estrogen, GABA-A modulators, Hormone receptor agonists, SGLT-2 inhibitors |
| Overall obesity-related cancer | 5 years | male | 0.989 (0.978,1.001) | 0.074 | 0.07 | 648/49435 | Type 2 Diabetes status, Cardiovascular disease, Chronic pancreatitis, Chronic kidney disease / ESRD, Essential hypertension, Heart failure, Musculoskeletal pain, Loop diuretics, Serotonin agonists, SGLT-2 inhibitors, Unknown cellular/molecular interaction |
| Overall obesity-related cancer | 10 years | female | 0.992 (0.981,1.004) | 0.23 | 0.18 | 314/30009 | Chronic pancreatitis, Musculoskeletal pain, Adrenergic alpha antagonists, Dopamine antagonists, GABA-A modulators, GLP-1 receptor agonists, Thiazide diuretics |
| Overall obesity-related cancer | 10 years | male | 0.991 (0.975,1.007) | 0.32 | 0.27 | 229/27896 | Type 2 Diabetes status, Chronic pancreatitis, Heart failure, Loop diuretics |
| Overall malignant cancer | 3 years | female | 0.993 (0.988,0.998) | 0.005 | 0.004 | 3651/52851 | Type 2 Diabetes status, Cardiovascular disease, Cerebrovascular disease, Chronic pancreatitis, Essential hypertension, Heart failure, Musculoskeletal pain, NAFLD/NASH, Osteoarthritis, Pancreatitis, Biguanides (Metformin class), Dopamine antagonists, Estrogen, Estrogen-progestin combination, GABA-A modulators, GLP-1 receptor agonists, Histamine H1 receptor antagonists, Hormone receptor agonists, Loop diuretics, Progesterone, SGLT-2 inhibitors, Thiazolidinediones (TZD) |
| Overall malignant cancer | 3 years | male | 0.985 (0.979,0.991) | < 0.001 | < 0.001 | 4917/46038 | Type 2 Diabetes status, Cardiovascular disease, Chronic pancreatitis, Chronic kidney disease / ESRD, Depression, Essential hypertension, Heart failure, Musculoskeletal pain, NAFLD/NASH, Sciatica and low back pain, GABA-A modulators, GLP-1 receptor agonists, Hormone receptor agonists, Insulin, Loop diuretics, SGLT-2 inhibitors, Sulfonylureas, Thiazolidinediones (TZD) |
| Overall malignant cancer | 5 years | female | 0.993 (0.988,0.998) | 0.006 | 0.005 | 2758/48055 | Type 2 Diabetes status, Acute kidney failure, Cardiovascular disease, Cerebrovascular disease, Chronic pancreatitis, Chronic kidney disease / ESRD, Essential hypertension, Heart failure, Musculoskeletal pain, Biguanides (Metformin class), Estrogen-progestin combination, GABA-A modulators, GLP-1 receptor agonists, Hormone receptor agonists, Serotonin agonists, SGLT-2 inhibitors, Thiazide diuretics, Thiazolidinediones (TZD) |
| Overall malignant cancer | 5 years | male | 0.988 (0.983,0.994) | < 0.001 | < 0.001 | 3725/42033 | Acute kidney failure, Cardiovascular disease, Chronic pancreatitis, Chronic kidney disease / ESRD, Depression, Essential hypertension, Heart failure, Musculoskeletal pain, NAFLD/NASH, ACE inhibitors, GABA-A modulators, GLP-1 receptor agonists, Hormone receptor agonists, Insulin, SGLT-2 inhibitors, Sulfonylureas, Thiazolidinediones (TZD) |
| Overall malignant cancer | 10 years | female | 0.992 (0.986,0.998) | 0.013 | 0.011 | 1141/26764 | Acute myocardial infarction, Cerebrovascular disease, Chronic pancreatitis, Essential hypertension, Heart failure, Musculoskeletal pain, Pancreatitis, GABA-A modulators, GLP-1 receptor agonists, SGLT-2 inhibitors, Thiazide diuretics, Thiazolidinediones (TZD) |
| Overall malignant cancer | 10 years | male | 0.99 (0.982,0.997) | 0.007 | 0.005 | 1367/23260 | Acute myocardial infarction, Cardiovascular disease, Chronic pancreatitis, Chronic kidney disease / ESRD, Heart failure, Musculoskeletal pain, NAFLD/NASH, GABA-A modulators, GLP-1 receptor agonists, Loop diuretics, SGLT-2 inhibitors, Sulfonylureas, Thiazolidinediones (TZD) |

Corrected p-values were calculated using false discovery rate (FDR) adjustment and represent the primary basis for the statistical inference. Uncorrected p-values are reported solely for transparency and descriptive purposes; no conclusion should be drawn from them.

| **Table S9. Unweighted Model Details (Overall Obesity-related Cancer & Overall malignant cancer)** | | | | | |
| --- | --- | --- | --- | --- | --- |
| **Cancer** | **Year Cohort** | **Estimate** | **Corrected p-value** | **Uncorrected p-value** | **Additional Covariates** |
| Overall obesity-related cancer | 3 years | 0.99 (0.985, 0.995) | < 0.001 | < 0.001 | Type 2 Diabetes status, Cardiovascular disease, Cerebrovascular disease, Chronic pancreatitis, Chronic kidney disease / ESRD, Essential hypertension, Heart failure, Migraine / tension / cluster headaches, Musculoskeletal pain, NAFLD/NASH, Estrogen, GABA-A modulators, GLP-1 receptor agonists, Hormone receptor agonists, Loop diuretics, SGLT-2 inhibitors, Thiazolidinediones (TZD) |
| Overall obesity-related cancer | 5 years | 0.989 (0.983, 0.994) | < 0.001 | < 0.001 | Type 2 Diabetes status, Cardiovascular disease, Cerebrovascular disease, Chronic pancreatitis, Chronic kidney disease / ESRD, Essential hypertension, Heart failure, Musculoskeletal pain, ACE inhibitors, Adrenergic alpha antagonists, ARB, Estrogen, Estrogen-progestin combination, GABA-A modulators, GLP-1 receptor agonists, Hormone receptor agonists, Loop diuretics, SGLT-2 inhibitors, Thiazolidinediones (TZD) |
| Overall obesity-related cancer | 10 years | 0.992 (0.984, 1) | 0.055 | 0.050 | Type 2 Diabetes status, Cardiovascular disease, Cerebrovascular disease, Chronic pancreatitis, Chronic kidney disease / ESRD, Heart failure, Musculoskeletal pain, Adrenergic alpha antagonists, Dopamine antagonists, GABA-A modulators, GLP-1 receptor agonists, Loop diuretics |
| Overall malignant cancer | 3 years | 0.992 (0.989, 0.995) | < 0.001 | < 0.001 | Type 2 Diabetes status, Acute kidney failure, Acute stroke, Cardiovascular disease, Chronic pancreatitis, Chronic kidney disease / ESRD, Depression, Essential hypertension, Heart failure, Migraine / tension / cluster headaches, Musculoskeletal pain, NAFLD/NASH, Sciatica and low back pain, Anti-obesity medications, Biguanides (Metformin class), GABA-A modulators, GLP-1 receptor agonists, Histamine H1 receptor antagonists, Hormone receptor agonists, Insulin, Loop diuretics, Phentermine, Serotonin uptake inhibitors, SGLT-2 inhibitors, Sulfonylureas, Thiazide diuretics, Thiazolidinediones (TZD) |
| Overall malignant cancer | 5 years | 0.993 (0.990, 0.996) | < 0.001 | < 0.001 | Type 2 Diabetes status, Acute kidney failure, Acute myocardial infarction, Acute stroke, Cardiovascular disease, Chronic pancreatitis, Chronic kidney disease / ESRD, Depression, Essential hypertension, Heart failure, Musculoskeletal pain, NAFLD/NASH, Sciatica and low back pain, ACE inhibitors, Anti-obesity medications, Biguanides (Metformin class), Estrogen-progestin combination, GABA-A modulators, GLP-1 receptor agonists, Hormone receptor agonists, Loop diuretics, Serotonin uptake inhibitors, SGLT-2 inhibitors, Statins, Sulfonylureas, Thiazide diuretics, Thiazolidinediones (TZD) |
| Overall malignant cancer | 10 years | 0.991 (0.987, 0.995) | < 0.001 | < 0.001 | Type 2 Diabetes status, Acute myocardial infarction, Acute stroke, Cardiovascular disease, Chronic pancreatitis, Chronic kidney disease / ESRD, Eating disorders, Essential hypertension, Heart failure, Musculoskeletal pain, Sciatica and low back pain, Biguanides (Metformin class), GABA-A modulators, GLP-1 receptor agonists, Loop diuretics, Serotonin receptor interactions, SGLT-2 inhibitors, Sulfonylureas, Thiazolidinediones (TZD) |
| Corrected p-values were calculated using false discovery rate (FDR) adjustment and represent the primary basis for the statistical inference. Uncorrected p-values are reported solely for transparency and descriptive purposes; no conclusion should be drawn from them. | | | | | |
